# A Unified Multi-Functional Dynamic Spectrum Access Framework: Tutorial, Theory and Multi-GHz Wideband Testbed

**DOI:** 10.3390/s90806530

**Published:** 2009-08-21

**Authors:** Robert Qiu, Nan Guo, Husheng Li, Zhiqiang Wu, Vasu Chakravarthy, Yu Song, Zhen Hu, Peng Zhang, Zhe Chen

**Affiliations:** 1 Department of Electrical and Computer Engineering, Center for Manufacturing Research, Tennessee Technological University, Cookeville, TN 38505, USA; E-Mails: nguo@tntech.edu (N.G.); ysong21@tntech.edu (Y.S.); Vasu.Chakravarthy@wpafb.af.mil (V.C.); zhu21@tntech.edu (Z.H.); pzhang21@tntech.edu (P.Z.); zchen42@tntech.edu (Z.C.); 2 Department of Electrical Engineering and Computer Science, The University of Tennessee Knoxville, TN 37996-2100, USA; E-Mail: husheng@eecs.utk.edu; 3 Department of Electrical Engineering, Wright State University, 3640 Colonel Glenn Highway, Dayton, OH 45435-0001, USA; E-Mail: zhiqiang.wu@wright.edu

**Keywords:** cognitive radio, testbed, cognitive radar

## Abstract

Dynamic spectrum access is a must-have ingredient for future sensors that are ideally cognitive. The goal of this paper is a tutorial treatment of wideband cognitive radio and radar—a convergence of (1) algorithms survey, (2) hardware platforms survey, (3) challenges for multi-function (radar/communications) multi-GHz front end, (4) compressed sensing for multi-GHz waveforms—revolutionary A/D, (5) machine learning for cognitive radio/radar, (6) quickest detection, and (7) overlay/underlay cognitive radio waveforms. One focus of this paper is to address the multi-GHz front end, which is the challenge for the next-generation cognitive sensors. The unifying theme of this paper is to spell out the convergence for cognitive radio, radar, and anti-jamming. Moore’s law drives the system functions into digital parts. From a system viewpoint, this paper gives the first comprehensive treatment for the functions and the challenges of this multi-function (wideband) system. This paper brings together the inter-disciplinary knowledge.

## 1. Introduction

In the most general sense, cognitive radio takes advantage of the Moore’s law to capitalize on the computational power of the semiconductor industry. When information is accessible in digital domain, the force driver behind this novel radio is computationally intelligent algorithms. Machine learning and artificial intelligence have become the new frontier toward this vision—analogy of robotics. Converting information from analog domain to digital domain plays a central role in this vision: revolutionary compressed sensing is, therefore, critical to expanding the territory of this new system paradigm. The agile, software defined radios that can perform according to algorithms are basic building blocks. When each node is computationally intelligent, wireless networking faces a novel revolution. At the system level, functions such as cognitive radio, cognitive radar and anti-jamming (even electronic warfare) have no fundamental difference and are unified into a single framework that requires inter-disciplinary knowledge. Radar and communications should be unified since both require dynamic spectrum access—the bottleneck. Spectrum agile/cognitive radio is a new paradigm in wireless communications—a special application of the above general radio.

**Figure 1.**
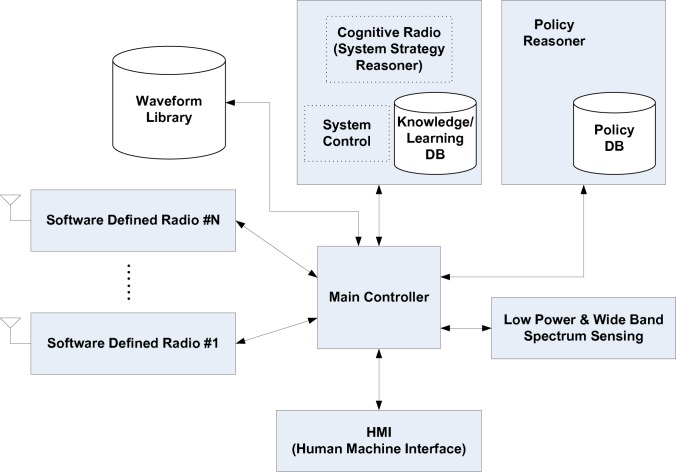
DARPA XG cognitive radio.

Spectrum agile/cognitive radio is a new paradigm in wireless communications [1], as illustrated by DARPA XG radio [2] in Figure 1. Cognitive radios can opportunistically use spectrum white space and increase usage by ten times [3]. One ingredient of this paper is to investigate a novel, wideband (multi-GHz) system architecture enabled by compressive sampling (or compressed sensing)—a revolutionary breakthrough in applied mathematics and signal processing. The other is to design multi-GHz spectrum sensing and experimental system testbeds. These ingredients share the same goal of bringing together three separate system paradigms: cognitive radio, cognitive radar and electronic warfare.

**Figure 2.**
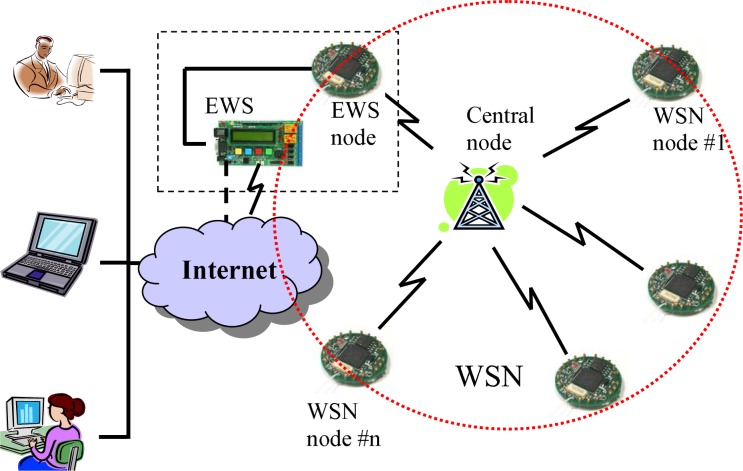
Embedded web server (EWS) for wireless sensor networks (WSN).

The Department of Defense (DoD) is transforming the military into a more responsive digitized force capable of of rapidly deploying and effectively operating in all types of military operations, which makes an intensive information network critical [4, 5, 6]. Wireless sensor networks in Figure 2 is such an example [7]. A 2003 Congressional Budget Office report [8] concluded: “current demand within the Army is larger than the supply by *an order of magnitude* and these shortfalls will continue into and after 2010 with shortage as high as *30 times* at some command levels.” To solve this bandwidth shortage, improvements in spectrum usage are required. These bandwidth shortages take place even though a vast amount of the allocated spectrum is virtually unused or under-used. This paradox results from the current static and inefficient allocation process. In response, the Federal Communication Commission (FCC) [9] and US DoD [10] recently issued separate challenges to address the poor efficiency of static spectrum assignment in licensed bands.

A recent study conducted by Shared Spectrum shows that average spectrum occupancy in the frequency band from 300 MHz to 3000 MHz over multiple locations is merely 5.2 %. The maximum occupancy is about 13% in New York City [11, 12]. It can be found that the spectrum scarcity is mostly caused by the fixed assignment to the wireless service operators, and there exist spectrum opportunities both spatially and temporally. Therefore, the interest in allowing access to unutilized spectrum by unlicensed user (second user) has been growing in several regulatory bodies and standardization groups, e.g., the FCC and IEEE 802.22—the first complete cognitive radio-based international standard [13].

In particular, the spectrum scarcity is the most severe problem for US for wireless services, partially due to the fact that US has the densest spectrum usage. There is a common belief that we are running out of usable radio frequencies. Cognitive radio (CR) provides an alternative (a new paradigm) to systems such as the third generation (3G) and the fourth generation (4G). As a result of the Department of Defense (DoD) focusing on the Joint Tactical Radio System (JTRS), US has a clear technical leadership in cognitive radio.

Cognitive radar [14], on the other hand, has similar demand for dynamic spectrum sharing. It is our conviction that it is, indeed, feasible to build a cognitive radar system using today’s technology. The advent of (multi-GHz) arbitrary waveform generators has made it possible to change waveforms from pulse to pulse [15]. Until recently, sensor hardware was not capable of changing the transmitted waveform in real time. We believe that the sensor hardware can be leveraged by jointly considering wideband spectrum sensing and waveform design.

Anti-jamming—an example of electronic warfare—is critical. The multi-GHz wideband platform proposed for both cognitive radio and cognitive radar may be further leveraged by including anti-jamming strategy (e.g., frequency hopping): it is much harder to jam the multi-GHz wideband communication and radar, compared with their multi-MHz counterparts. Our proposed experimental platform is one of the first of such integrated platforms.

There are two frequency bands where the cognitive radios might operate in the near future [11, 13]: 54–862 MHz (VHF and UHF TV bands) and 3–10 GHz (Ultra-wideband (UWB) radios) [16]. The FCC has noted that in the lower UHF bands almost every geographic area has several unused 6 MHz-wide TV channels. In 2002, the FCC approval of UWB underlay networks in 3–10 GHz indicates that this frequency range might be opened for opportunistic use.

Since CR uses opportunistic transmission, it is desirable to operate over the widest possible bandwidth to give the highest probability of detecting unused spectra [17]. The unique sensing function forces the front-end to have several GHz sampling rate with high resolution (of 12 or more bits), if GHz bandwidths are to be searched [18]. One of the most demanding challenge is posed by wide bandwidth: make UWB RF front-end able to access spectrum dynamically.

There exist two basic problems for system concepts: (1) How do we deal with baseband signals of several GHz bandwidth, say 3 GHz (0–3 GHz) or 7.5 GHz (3.1–10.6 GHz)? (2) How do we handle the dynamic range of spectrum sensing over the bandwidth of several GHz? The objective of this paper is to address these two problems.

The system is designed using a revolutionary new theory, also known as compressed sensing [19, 20, 21]. By exploiting the *structure* of the natural signal, a sampling rate that is much lower than the Shannon/Nyquist rate can be used to recover the “information” of the analog signal with overwhelming probability. We have demonstrated a UWB system baseband bandwidth (5 GHz) that would take decades for the industry to reach with the conventional sampling technology. We could use standard converters at the level of 125 megasamples per second (MS/s), for which excellent high dynamic range commercial solutions are available—a big advantage of the proposed approach.

UWB radios are revolutionary due to its unprecedented bandwidth—three orders of magnitude higher than the typical wireless systems. Their signals exhibit many unique properties such as transient and impulsiveness—they are sparse in some domain (e.g., time). The sparseness—the very fundamental notion underlying compressed sensing—can be exploited to reduce sampling rate. Compressed sensing framework provides a universal measurement approach for signal detection and estimation, without reconstructing the signal—a quasi-digital receiver. Unlike the analog-intensive correlation receivers (popular for UWB), extremely wideband analog delay element is not required.

The fact that space-time signals are essentially always significantly compressible in some representation promises huge benefits. These compressive sampling protocols are noteworthy for the relatively limited prior knowledge about the class of the signal to be acquired: basically just the knowledge that the signal of interest would be compressible within a certain representation—theoretically demonstrated, for a class of UWB radio and acoustic signals, by the first author recently [22]. These classes are quite large and, in principle, one compressive sampling protocol works for the whole class. This paper focus on the rigorous determination of the potential impact of these and other fundamental research concepts on practical communications approaches. The proposed research will uncover significant opportunities and establish various important bounds on the sampling required, as a function of prior and ancillary information, about the RF environment and the particular application (UWB cognitive radio).

## 2. Summary of the Paper

**Figure 3.**
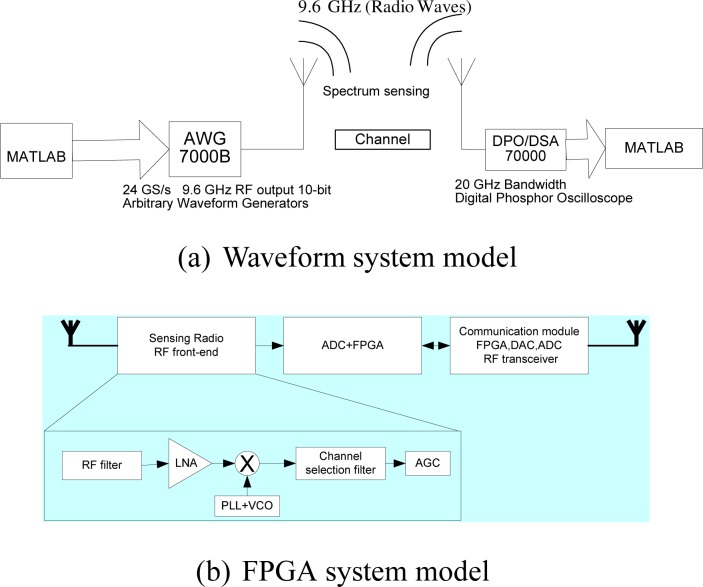
Experimental systems.

The objective is to seamlessly integrate (into a single platform) three system ingredients: cognitive radio, cognitive radar and anti-jamming. One primary task of this proposed research is to provide new analytical and computational tools to allow for practical implementation of compressed sensing and, eventually, to aid the design of sensors that are capable of carrying out direct measurements motivated by the established theoretical bounds. Radar is a remote sensing system well suited for cognition [14]. The knowledge-based cognition is inherent in cognitive radio and radar, and can therefore be jointly designed together with anti-jamming. For example, waveform designs for radar sensing [15] and cognitive radio [23, 24] can, indeed, be co-designed in the same framework. Both underlay scheme (e.g., UWB radio IEEE 802.15.4a [16, 25, 26, 27]) and overlay scheme (e.g., TV band cognitive radio IEEE 802.22) can be studied in the unified framework for both cognitive radio and cognitive radar. Anti-jamming is the prerequisite for most DoD communication and radar applications, and can be naturally fitted into the unified framework. The focus is on the basics and proving the concept: multi-GHz waveforms and spectrum sensing, theoretical framework, and hardware testbed. The primary challenge is caused by the wideband (multi-GHz) nature of the problem at hand. Compressive sampling provides a new paradigm to greatly relax the ADC, which simplifies the front end. Compressed sensing can be also used to reduce the ADC for radar sensing [28]. On the other hand, wideband (multi-GHz) spectrum sensing—very challenging—is used for dynamic spectrum access. New anti-jamming capabilities can be explored in this novel framework. Experimental systems with real life imperfections are proposed Figure 3.

Below is a list of topics being discussed extensively in the following sessions:

- Spectrum Sensing for Cognitive Radio
- A Survey on Cognitive Radio Implementation Research
- Multi-Giga-Hertz Agile Radio Front-End Design
- A Compressed Sensing Based Ultra-Wideband Cognitive Radio
- Wideband Waveform Optimization for Multiple Input Single Output Cognitive Radio Using Time Reversal
- A Unified Framework for Cognitive Radio and Cognitive Radar
- Quickest Spectrum Sensing
- Soft Decision Cognitive Radio and Hybrid Overlay/Underlay Cognitive RadioWaveform Design

### 2.1. Compressed Sensing

Compressed sensing builds upon a core tenet of signal processing and information theory: that signals, images, and other data often contain some type of *structure* that enables intelligent representation and processing. For example, in imaging compressing, a correlated signal’s energy can be compacted into just a few essential coefficients. Such transform coders exploit the fact that many *natural* signals have a *sparse* representation in terms of some basis (e.g., Fourier, wavelet).

Fortunately, by the first author [22, 29, 30], transient electromagnetic signals and acoustic signals are rigorously shown to have such a structure, called compressibility. UWB signals (used for communications and radar) belong to this class of transient signals. This theoretical work paves the way for the practical application, since the prerequisite for using the CS principle is that channel impulse response is compressible in some basis, either frame or dictionary. Using singular value decomposition (SVD) —an orthogonal basis—of the channel impulse response, the first author has proved that the SVD coefficients follow a power-law decay and thus satisfy the definition of compressibility [22, 30]. The focus of this proposal is to demonstrate the real-world UWB system, designed by using a CS principle.

### 2.2. A Compressed Sensing Based Ultra-Wideband System

The concept of the compressed sensing based UWB system is illustrated in Figure 26. The core of the system lies in compressed sensing [19, 20, 21, 31]. For example, for an RF bandwidth of 5 GHz, a sampling rate of 125 MHz is sufficient for a measured channel impulse response indoors. This example has demonstrated the power of the CS principle to reduce the receiver complexity by simplifying the mixed signal processing. Related concept is used in radar [28] and imaging [32].

### 2.3. Wideband (Multi-GHz) Spectrum Sensing

There are two classes of cognitive radios: (1) narrowband cognitive radio; (2) wideband cognitive radio. This classification is purely for the convenience of description in the context of spectrum sensing. Two motivating examples are given to illustrate this fundamental difference: (1) 6 MHz cognitive radio for unlicensed digital TV band—IEEE 802.22 [33]; (2) wideband tunable (over several GHz bandwidth) cognitive radio—evolution beyond 802.22.

The narrowband cognitive radio—band specific (TV band)—is much easier for spectrum sensing. It can exploit *a prior* information about one particular system configuration, such as air interface, modulation format, symbol rate, pilot, etc. Its wideband counterpart generally has not so much information to use. Spectrum is like disk space, the more one has, the more applications that will use it [3]. The front-end, including A/D, filters, wideband and real-time sensing, is the primary challenge for the wideband case. Spectrum sensing for the physical layer design is another challenge [3]. The wideband CR is the primary focus of this proposed research. We will systematically evaluate the 802.22 technologies of the narrowband CR [3, 34, 35, 36, 37, 38] before their use in the wideband CR.

Spectrum sensing is one of the major challenges for cognitive radio, since the signal is weak to detect and protect (at low SNRs) [36, 39]. For example, TV broadcasters have set a stringent limit for the digital TV signals to be reliably detected (probability of detection > 90% with probability of false alarm > 10%) at a signal strength of 116 dBm translating to roughly −21 dB of signal-to-noise ratio (SNR) based on the receiver noise figure (NF) of around 11 dB and the use of omnidirectional antenna for spectrum sensing [6].

### 2.4. Waveform System Model and FPGA System Hardware Testbed

Wideband (multi-GHz) cognitive radio is in its infancy, compared with its narrowband counterpart IEEE 802.22. It is critical to test key system components in different system settings. Three system models are proposed: (1) MATLAB/C simulation model; (2) Waveform model; (3) Real-time FPGA system model.

Most research is carried out in the domain of MATLAB/C simulation model. This approach is simple. But many real-world limitations cannot be simulated. The unique approach of this proposal is to combine these three models. Real-time FPGA model (Figure 3(b)) is the ultimate test, but time-consuming. We will use this model when the system is very stable. As a result, most system emulations are based on the waveform model in Figure 3(a). A waveform of 9.6 GHz effective RF bandwidth with 10-bit resolution can be transmitted over the air, and captured after transmission. This system produces high-speed serial waveforms with real life imperfections including noise, jitter, pre/de-emphasis, and multi-level signaling up to 8 Gb/s. This waveform model is made available only recently (to the best knowledge of the first author, this is available less than one year), with the latest A/D conversion. TTU’s lab is fortunate to have the NSF MRI grant to make this possible.

### 2.5. Challenges of the Wideband Front End

The main components of a cognitive radio transceiver are the radio front-end and the baseband processing unit. Each component can be reconfigured via a control bus to adapt to the time-varying RF environment. In the RF front-end, the received signal is amplified, mixed and A/D converted. In the baseband processing unit, the signal is modulated/demodulated and encoded/decoded. The baseband processing unit of a cognitive radio is essentially similar to existing transceivers. However, the novelty of the cognitive radio is the RF front-end. So we will first focus on the RF front-end of the cognitive radios [40].

For wideband (multi-GHz) cognitive radio, there may be interfering signals that are much stronger than the CR signal of interest, resulting in signal to interference ratios as low as −50 dB. This requires a large dynamic range for the front-end circuitry and in particular for the ADC which must accommodate the large interfering signals while still provides sufficient quantization performance for the weak CR signal [18]. In typical cognitive radio sensing scenario, the RF signal presented at the antenna includes signals from closely and widely separated transmitters, and from transmitters operating at widely different power levels and channel bandwidths. As a result, the large dynamic range becomes the main challenge as it sets the stringent requirements on circuit linearity and resolution of A/D converters [11, 41].

Generally, a wideband front-end architecture of the cognitive radio has the following structure (Figure 3(b)). The components of a cognitive radio RF front-end are as follows: (1) RF filter: the RF filter selects the desired band by bandpass filtering the received RF signal. (2) Low noise amplifier (LNA): the LNA amplifies the desired signal while simultaneously minimizing noise component. The LNA should have minimal noise figure (e.g., 2–3 dB) in order to have good sensitivity at low power. (3) Mixer: in the mixer, the received signal is mixed with locally generated RF frequency and converted to the baseband or the intermediate frequency (IF). The mixers have to maintain the linearity across entire dynamic range and bandwidth. However, these are also conflicting requirement with respect to power consumption. (4) Voltage-controlled oscillator (VCO) and phase locked loop (PLL): the VCO and PLL need to quickly generate a signal at a specific frequency with fine resolution. This is a key challenge for wideband spectrum sensing. (5) Channel selection filter: The channel selection filter is used to select the desired channel and to reject the adjacent channels. (6) Automatic gain control (AGC): The AGC maintains the gain or output power level of an amplifier constant over a wide range of input signal levels.

Furthermore, large dynamic range and sampling of wideband signals further require high precision and high speed A/D converters. Unfortunately, the design of high speed A/D converters has fundamental limits in terms of achievable resolution. The requirement of a multi-GHz speed A/D converter necessitates the dynamic range of the signal to be reduced before A/D conversion.

In summary, the key challenge of the physical architecture of the cognitive radio is an accurate detection of weak signals of licensed users over a wide spectrum range. Hence, the implementation of RF wideband front-end and A/D converter are critical issues that will be addressed in this paper.

### 2.6. Cognitive Radar and Anti-Jamming

Three ingredients are fundamental to the cognitive radar [14]: (1) intelligent signal processing, which builds on learning through interactions of the radar with the surrounding environment; (2) feedback from the receiver to the transmitter, which is a facilitator of intelligence; and (3) preservation of the information content of radar returns, which is realized by the Bayesian approach to target detection through tracking.

This cognitive cycle performed by a cognitive radar system—a two-way process—is similar to the time reversal communication system [42], in the spirit that the environment has a strong and continuous influence on the radar returns (i.e., multipath echoes). In doing so, the radar builds up its knowledge of the environment from one scan to another and make decisions of interest on possible targets at unknown locations in the environment. Unlike a communication system, the feedback mechanism, which is a necessary requirement of the cognitive system, is easier to implement, as the radar transmitter and receiver are usually co-located.

To simplify the description, we will use the cognitive radio as the motivating system; most of contents are, however, valid to cognitive radar. For anti-jamming (shared by both communication and radar), we emphasize the new possibility enabled by wideband (multi-GHz) front end. Hardware is our primary concern in the concept proof stage. New algorithms will be developed when more experimental results are available.

### 2.7. Significance and Related Work

For wideband (GHz) spectrum sensing, there is no practical way to locate all receivers of communications from the transmitter [43]. One challenge is wideband RF front-end capable of simultaneous sensing of several GHz wide spectrum [11, 17, 18, 41, 44, 45].

ADC implementations—trade sampling frequency against dynamic range—are the bottleneck of some emerging applications such as wideband cognitive radios and cognitive radar. Emerging applications require conversion of instantaneous bandwidth in the gigahertz range with dynamic range of up to 16 bits. This translates to ADC sampling rates of multiple gigasamples per second (GS/s) with a sample aperture jitter held to one-tenth of a picosecond. Current capabilities fall well short of needs and are advancing at a rather slow rate—improving about 1.5 bits in eight years. For example, UWB is allowed to operate from 3.1 to 10.6 GHz (a bandwidth of 7.5 GHz). ADC speed is far behind the need for digital receivers.

Compressed sensing principles enable the design of flexible imaging devices and techniques [32]. By time multiplexing a single detector, they can employ a less expensive and yet more sensitive photo sensor. Their new camera architecture that employs a digital micromirror array to perform optical calculations of linear projections of an image onto pseudo-random binary patterns. Its hallmark includes the ability to obtain an image with a single detector element, while sampling the image fewer times than the number of pixels. The idea is to off-load processing from data collection into data reconstruction. Not only will this lower the complexity and power consumption of the device, but it will enable adaptive new measurements schemes. The most intriguing feature of their system is that, since it relies on a single photo detector, it can be adapted to *image at wavelengths that are currently impossible with conventional imagers*.

This surprising feat of the single-detector camera has inspired this research. A natural question arises from this observation. Can one use the same principle for wideband (multi-GHz) communication and radar? The UWB signal at the receiver consists of short, transient pulses with huge bandwidth that is impossible for the current semiconductor industry to handle. The UWB signal, however, is *sparse* in time domain (like Dirac pulses). Can this signal sparsity be exploited in a novel UWB system design? This idea appears very promising, according to preliminary investigations. For example, a system bandwidth of 5 GHz has been achieved over the wireless channel, by using an A/D converter of 125 MHz (as described above). As in the case of the one-detector camera, the most important advantage is to adapt this system architecture to work at bandwidths that are currently impossible with conventional designs (based on Shannon/Nyquest sampling). For example, the most advanced A/D can sample at 10 GS/s with 8 bits (www.Maxtek.com), and D/A (model DX-10G) can reach a clock rate of 10 GS/s with 10 bits. Assuming analog processing is available at the transmitter, the system bandwidth can go much higher. This implies digital signal processing will, thus, be feasible at the receiver.

On the other hand, there is some sparsity in spectrum that can be exploited in the framework of CS [46]. This potential is not as large as the signal domain since we can pre-code the transmitted waveform in a way suitable for CS.

### 2.8. Quickest Detection for Spectrum Sensing

Spectrum sensing is the (instead of ‘a’) key problem in cognitive radio systems. Secondary users need to monitor the spectrum occupancy in order to use the spectrum when there is no primary user or quit the spectrum when primary users emerge. Like all other detection (or equivalently, hypotheses testing) problems, the spectrum sensing needs to find a tradeoff between miss detection (not detecting the primary user when it emerges) and false alarm (claiming that a primary user exists when there is actually no primary user). Due to the requirements of tolerable violation to primary users and tolerable interruption to the communication of secondary users, the spectrum sensing has substantial impact on the overall performance of cognitive radio systems.

**Figure 4.**
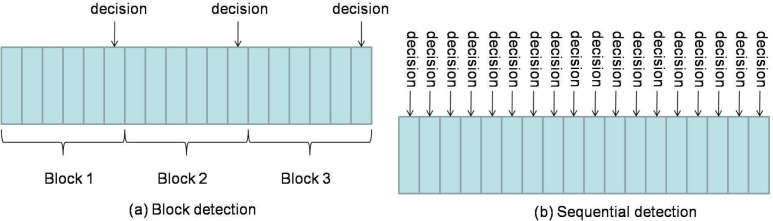
Illustrations of block and sequential detections.

In most existing publications on spectrum sensing, traditional block detection is used, in which the observations are grouped into blocks and decision is made at the end of each block based on the observations of the corresponding block (illustrated in Figure 4 (a)). The advantage of such a block detection is easy implementation. However, it is difficult to determine the size of each block: if the block size is small, the decision will be unreliable; if the block size is large, then the detection delay may be large (e.g., a primary user emerges at the beginning of a block and can be detected only at the end of the block; then the delay will be the block duration).

**Figure 5.**
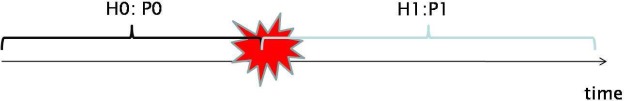
Illustration of quickest detection.

Therefore, a more suitable framework for spectrum sensing is sequential detection (illustrated in Figure 4 (b)), in which the decision could be made when each new observation arrive. Moreover, we notice that the observation distribution could change (e.g., the average received power is increased when primary user emerges), which is different from traditional detection problems (the distribution is static). Hence the *spectrum sensing is essentially to detect the change of observation distribution*. A powerful tool for such a problem of change detection is *quickest detection* (also called quickest change detection or abrupt change detection). As illustrated in Figure 5, we assume that the original observation distribution is *P*_0_ and the distribution is changed to *P*_1_ at a time unknown to the detector. The job of quickest detection is to detect the change as quickly as possible under the constraint of tolerable false alarm rate. We coin the spectrum sensing within the framework of quickest detection as *quickest spectrum sensing*.

Research on quickest detection dates back to 1931 [47] and has attracted substantial research since then. Quickest detection is useful in tasks like remote sensing [48, 49, 50], financial decision making [51, 52, 53, 54, 55], medical diagnosis [56, 57, 58, 59], signal segmentation [60, 61, 62, 63], environmental monitoring [64, 65], and network security [66, 67, 68, 69, 70, 71]. In recent years, decentralized quickest detection has received plenty of studies [72, 73, 74]. Comprehensive introductions on quickest detection can be found in [75] and [76].

The quickest spectrum sensing can be categorized according to the following criterions:

- Bayesian vs. non-Bayesian: the *a priori* probability of primary user activity is available [77] in the former case and unavailable in the latter case [78];
- Single channel vs. multiple channels: whether the secondary user could monitor one channel or multiple channels in the frequency spectrum;
- Completely observable vs. partially observable: this applies to only the case of multiple channels; in the former situation, the secondary user can get observations from all channels whereas it can monitor only a subset of channels in the latter case.

In the remainder of Section 9., we will discuss two typical scenarios of quickest spectrum sensing: single channel with non-Bayesian detection and multiple channels with Bayesian detection based on partial observations.

### 2.9. Overlay and Underlay Transmission for Cognitive Radio

In current cognitive radio, there are two approaches to use the spectrum more efficiently: the overlay cognitive radio transmission and the underlay cognitive radio transmission (UWB). In underlay CR, a very wide bandwidth is occupied by the transmission with a very low power spectrum density. This extremely low spectrum density of underlay CR transmission avoids significant interference to existing primary users operating in the range of the underlay CR transmission. In overlay cognitive radio, frequency agile transmitters discover unused spectrum “holes” and transmit over those unused frequency bands. By doing so, interference to existing wireless systems is avoided.

However, both underlay CR transmission and overlay CR transmission are not without drawbacks. In underlay CR, the transmission power is extremely limited in order to avoid interference to primary users, which significantly decreases the available channel capacity. In overlay CR, only unused frequency bands are exploited to transmit signal and all underused bands are not touched at all, which decreases the available bandwidth and channel capacity. In Section 10., we will address this issue.

## 3. Spectrum Sensing for Cognitive Radio

A CR dynamically alters its own frequency assignment after sensing its local spectrum and ultimately does not impact the performance of the primary network [4]. Physical (PHY) layer issues include spectrum sensing algorithms, low SNR signal detection, wideband or narrowband sensing, adaptive modulation and coding, waveform shaping, ADC, programmable filters. Medium access control (MAC) issues include coordination of quiet periods, spectrum sensing management, contiguous multichannel operation, inter-channel synchronization, real-time dynamic resource allocation, and multi-channel access.

### 

#### Spectrum Sensing for Narrowband Cognitive Radio

An exhaustive review of spectrum sensing in IEEE 802.22 is given in [39]. A good survey of cognitive radio is [40]. Energy detection, pilot detection and collaborative detection are experimentally studied in the context of DTV [79]. Dynamically selecting one out of six (1.75 MHz) channels within 225–600 MHz was field tried within DARPA’s XG program [80, 81]. Pilot-based sensing is used for fine sensing in IEEE 802.22, but is non-blind (ATSC -specific). A distributed approach is used [82].

##### Cyclostationary Sensing

The inherent spectral redundancy (in cyclostationary signals [83]) caused by the use of a cyclic prefix in OFDM signals may be exploited, first by [11, 44] and then [84, 85, 86]. A unified approach to the recognition of signals belonging to the three basis air interfaces categories: single carrier TDMA, OFDM systems, single carrier CDMA systems [87]. It is also used to WCDMA [88]. It has been used in a framework of overlay/underlay cognitive radio [23, 24].

This unified approach may be the most promising, if there is some *prior* information about communication such as modulation format [83]. Although valid for some commercial systems above, this is generally not true for DoD systems. Higher-order statistics (HOS) of the cyclostationary signals is needed.

##### Non-Gaussian Nature of the Frequency Domain Signals

No prior knowledge of the type of signals present in the spectrum is often known. Signal detection in Gaussian noise can be carried out using HOS [6]. The fact that the cumulants of the order higher than two for a Gaussian process are zero can be used to detect the signals in the Gaussian noise. The received waveform samples can be grouped into segments, and higher order cumulants for each of these segments can be estimated. The detection thresholds are defined after a period of learning the distributions of the moments and cumulants, and a decision is made whether a particular segment of the received samples contains meaningful information or not. This is a test (also known as bi-coherency, tri-coherency, etc.) to determine if the received waveform belongs to the DTV signal or noise. In the time domain, DTV signals show Gaussian characteristics. In the frequency domain, however, they are non-Gaussian. We will exploit such characteristics to detect the signals in AWGN.

#### Spectrum Sensing for Wideband Cognitive Radio

Wideband (e.g., from 0 to 3 GHz) spectrum sensors scanning multiple licensed bands may not be able to include all feature detection algorithms necessary to identify all incumbents operating in the measured band [43]. In this case, it may be preferred to use energy detection. Other wideband sensing techniques are needed.

At the cognitive radio (CR) transmitter, this sensing and transmission function is performed over the widest possible bandwidth to give the highest probability of detecting unused spectra—opportunistic transmission [17]. The unique sensing function forces the front-end to have several GHz sampling rate with high resolution (of 12 or more bits), if GHz bandwidths are to be searched [18].

The main limitation in a radio front-end’s ability to detect weak signals is its dynamic range, which dictates the requirement for number of bits in A/D converter. Since it is difficult to design high-resolution A/D converters—the pricing will not follow Moore’s law, it is highly desirable to relax the A/D requirement. Also, the power and A/D complexity rises almost exponentially with the number of bits.

There is a synergy between compressed sensing and spectrum sensing. The former enables the use of a bigger signal bandwidth to fully exploit the potential of the 7.5 GHz unlicensed spectrum allocated by FCC. The latter enhances the opportunity of the system to use unoccupied spectra.

Operation of WiMax in the 3.5 GHz band is susceptible to interference from UWB devices [89].

##### Energy-Based Sensing

The non-coherent energy based approach does not require *a prior* knowledge of the signal to detect, and results in far fewer calculations to reach decision, enabling a larger bandwidth to be surveyed at all times. The disadvantages are its lower sensitivity to the weak signal and the requirement for the adaptive threshold setting.

##### Eigenvalue-based sensing

The advantage of the autocorrelation matrix approach is to rely on the spectrum only. Using the Wiener-Khintchine theorem, the autocorrelation estimate at lag *τ* in the band of interest defined by *ω_m_*, *m* ∈ [1, *M*] is given by [4]

(1) $$R_{\mathrm{xx}}\left(\tau_{n}\right)=\frac{2}{M}\;\sum_{m=1}^{M}{\left|S\left(\omega_{m}\right)\right|}^{2}\;e^{j2\pi \omega_{m}\tau_{n}}$$

where $\tau_{n}=n\delta t=\frac{n}{F_{s}}$ with *δt* the sampling period equal to the inverse of the sampling frequency *F_s_*. A a result, only the spectrum is used. In other words, the coherent information or phase has been removed. Once the autocorrelation is collected, its matrix can be formed and its eigenvalues can be used to detect the unused/under-used spectrum [90].

#### Measurement Systems for Sensing

**Figure 6.**
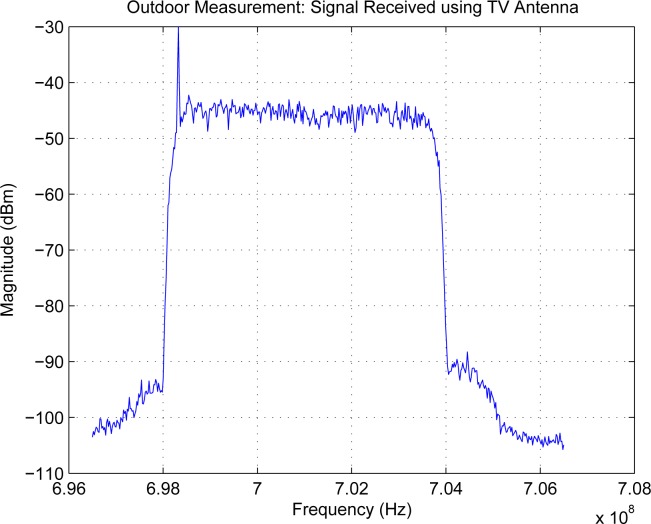
Digital TV Signal. Pilot is noticed in the left.

Measurements of spectrum for interference temperature are basic to cognitive radio, as is channel sounding to wireless communications. A measurement system using spectrum analyzer controlled by Labview has been developed for 9 kHz–26.5 GHz—Figure 6 is a sample of collected data. The delay for each snapshot of the spectrum is in the level of 40 ms.

Since sensing window is in the level of 5–25 ms for IEEE 802.22 [34, 35, 36, 38, 39], much quicker measurements are required. For this purpose, we will use real-time spectrum analyzer Tektronix RSA 6114A. Multiple correlated views are available in all domains (frequency, time, amplitude, phase, modulation). The statistics of the spectrum will be essential to future system designs. For example, quickest detection [91] is a framework to incorporate the statistics into the algorithms for wideband (several GHz) spectrum sensing [92].

#### Preliminary Results and Proposed Tasks

Roughly speaking, TTU’s Lab has developed the necessary equipment infrastructure for long-term research in the area of wideband cognitive radio. The measurement equipment is available for spectrum statistics study. Proposed tasks are to answer the following questions: (1) What are the long-term and short-term statistics of the wideband spectra? (2) How does the statistics change with the geo-location? (3) What algorithms are suitable for wideband spectrum sensing? (4) How is the statistics incorporated into algorithms?

How does a CR select the best portions of the spectrum to use [4]? What amount of spectrum will a CR be able to harvest in urban/sub-urban areas? How can a CR limit the risk of using spectrum that only appears to be unused locally but is indeed being used nearby (hidden-node problem)?

## 4. A Survey on Cognitive Radio Implementation Research

For cognitive radio, ultimately, the ability to reliably recognize the communication environment and agilely adapt the transmission parameters to maximize the quality of service (QoS) while minimizing the interference to the primary users can only be addressed and justified by real working systems [93]. However, for now it is far from clear what mechanisms are best suited to implement cognitive radios, both with respect to preventing interference and with respect to efficiency and performance. There are a plethora of techniques (cooperative sensing, cyclostationary detectors, Higher Order Statistics Sensing, etc.) that have been proposed to enhance detection. None of these techniques have been tested in real world scenarios and their performance has yet to be characterized [94]. Thus, the cognitive radio implementations research becomes a significantly important part in this area.

Currently, there are a bunch of testbeds/platforms have been developed and used for cognitive radio experiments by industry and academia. A subset of most recent platforms that are mostly widely available will be covered in this paper.

### 4.1. GNU/USRP Radio Platform

**Figure 7.**
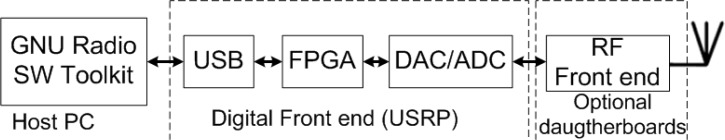
Architecture of GNU software Radio.

GNU/USRP radio is one of the largest scale open source software defined radio platform today [95, 96]. It consists of GNU software package and the USRP hardware platform, Figure 7 shows a typical block diagram for a the GNU/USRP radio. The GNU radio is a free/open-source software toolkit for building software radios, in which software defines the transmitted waveforms and demodulates the received waveforms. The USRP (Universal Software Radio Peripheral) is the associated hardware platform, which is completely open to the public, including the circuit schematic and FPGA source code [95, 96].

With GNU radio’s open architecture, open source code, available functionalities developed by developers from all over the world grows quickly. Because it is based on general purpose processor (GPP) architecture, it is flexible, extensible and portable. Currently, the GNU radio software capabilities support the development of various waveforms including AM and FM analog waveforms, narrowband digital waveforms of GMSK, BPSK, QPSK, and even multi-carrier waveforms [95]. GNU radio is written in both C++ and Python language, and programs can be compiled and run on most GPPs and operating systems including Linux, Mac OSX, and Windows XP. Typically GNU radio is used with a USRP radio front-end.

**Figure 8.**
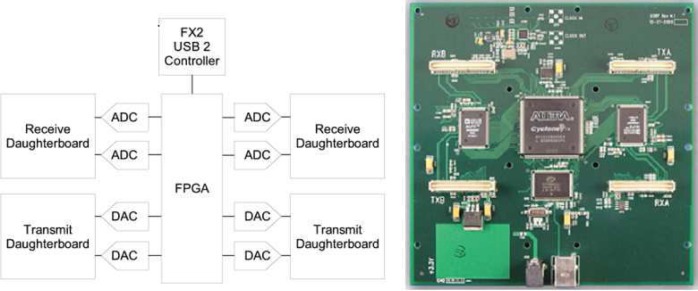
USRP architecture.

The USRP, whose architecture and layout are as described in Figure 8, consists of a small motherboard containing up to four 12-bit 64M sample/s ADCs for receive functions, four 14-bit, 128M sample/s DACs for transmit functions, a million gate Altera Cyclone FPGA and a programmable USB 2.0 controller. Each fully populated USRP motherboard supports four daughterboards, two for receiving and two for transmission. RF front ends are implemented on the daughterboards. Depending on the specific daughterboards added, it can cover a variety of frequency bands. A list of current available daughterboards with specification is shown in Table 1 [96].

Hardware drivers for the USRP are included in the standard build of GNU radio software package by default, most of the USRP settings, such as center frequency, PGA gain, interpolation, decimation, and other transmission and receiving path options on the USRP can be controlled using GNU Radio. The drivers for the USRP in the GNU radio package are provided both at elementary C++ class level and Python API function level [96].

GNU/USRP radio provides great flexibility to support various independent cognitive radio developments through software. However, it cannot support high computational throughput for real-time processing and controlled physical and network layer integration.

### 4.2. Cognitive Radio Platform from UC Berkeley

The BWRC (Berkeley Wireless Research Center) cognitive radio testbed hardware architecture consists of Berkeley Emulation Engine (BEE2) [97], reconfigurable 2.4 GHz radio modems, and fiber link interface for connection between BEE2 and radios. The software architecture consists of Simulink-based design flow and BEE2 specific operating system that provide an integrated environment for implementation and simple data acquisition during experiments [94].

The BEE2 contains 5 Vertex-2 Pro FPGAs, each FPGA embeds a PowerPC 405 core, which minimizes the latency between the microprocessor and reconfigurable logic while maximizing the data throughput. Furthermore, with FPGAs running at clock rates similar to that of the processor cores, system memory, and communication subsystems, all data transfers within the system have tightly bounded latency. BEE2 is therefore well suited for high throughput real-time applications [97].

**Figure 9.**
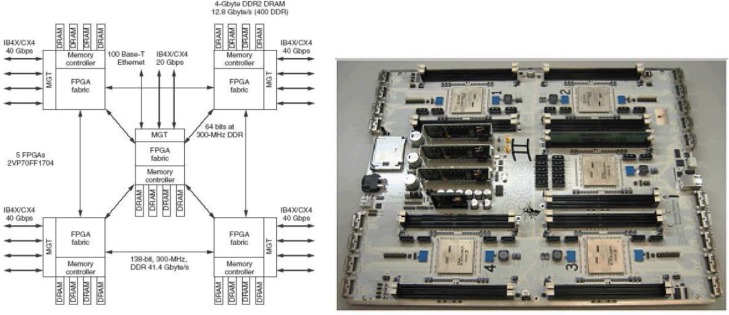
BEE2 system architecture (left) and BEE2 implementation (right).

In order to interface this real-time processing engine with radios and other high throughput devices, multi-gigabit transceivers (MGTs) on each FPGA are utilized together with physical XAUI 4X electrical connection to form a 10 Gbps full-duplex links. There are a total of 18 such interfaces per BEE2 board allowing independent connections of 18 radios. Each individual MGT channel is software configurable to communicate and exchange data at any rate below 10 Gbps. The board also contains USB and JTAG interfaces. Figure 9 shows the architecture and a picture of the BEE2 board. Details about BEE2 can be found in [97].

The reconfigurable wireless modem consists of the filters, ADC/DAC chips and a Xilinx Vertex-II Pro FPGA. Digital-to-analog conversion is performed by a 14-bit DAC running at 128 MHz, while analog-to-digital conversion is performed by a 12-bit ADC running up to 64 MHz. The FPGA performs data processing and control, and supports 10 Gbps full duplex XAUI link for transmitting and receiving data to/from BEE2. The RF modem module is capable of up/down converting 20 MHz RF bandwidth at 2.4 GHz. The RF frequency is fully programmable in the entire 80MHz ISM band using LMX2326 synthesizer [98].

**Figure 10.**
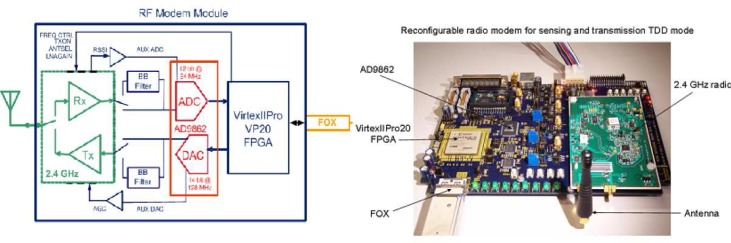
The BWRC Reconfigurable wireless modem.

Top level block diagram and implementation of the wireless modem are presented in Figure 10. Both received signal strength (RSSI) and automatic gain control (AGC) are measured in real-time to support optimal signal conditioning on the receiver end. It also features dual antenna configuration for switched antenna diversity [98].

Software design is built around Matlab/Simulink from Mathworks [99] coupled with the Xilinx System Generator [100] for mapping high-level block diagrams and state machine specifications to FPGA configurations. This environment supports simultaneous development of signal processing algorithms and digital design description for their hardware realization. Therefore no translation is required and allows signal processing researchers to realize hardware implementation of developed algorithms [94, 98, 101].

**Figure 11.**
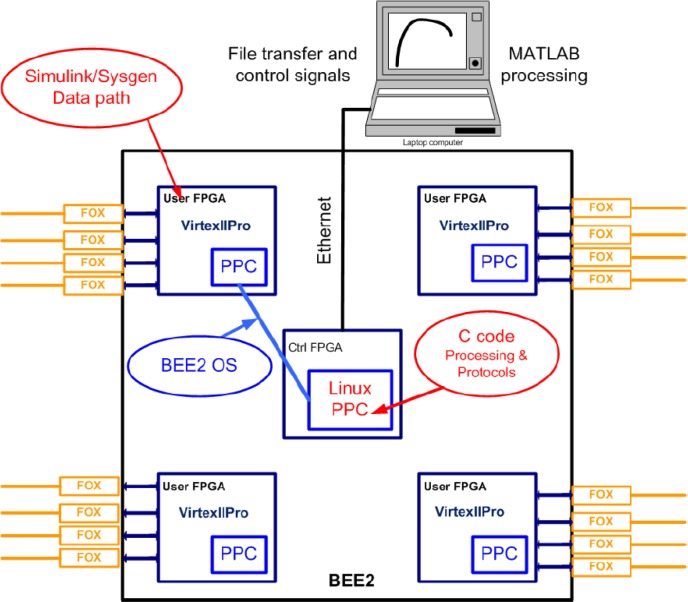
Software design flow for mapping of algorithms and protocols on BEE2.

One of the key features in the design flow is the ability to communicate and control hardware registers, block RAMs, DRAMs, and software running on control FPGA in real-time. BEE2 can be connected to the local area network, so that registers and memory can be accessed and transferred to laptops or PCs via Ethernet. Figure 11 illustrates the mapping process of algorithms and protocols on BEE2 as well as experiment control via Ethernet [98, 101].

### 4.3. ORBIT Platform from Rutgers University

**Figure 12.**
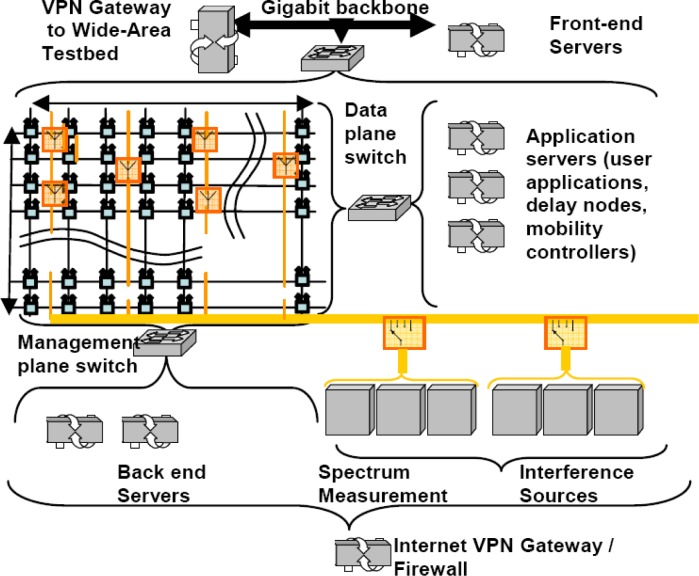
Orbit system architecture.

The Orbit (Open Access Research Testbed for Next-Generation Wireless Networks) testbed developed by WinLab at Rutgers University is a large-scale wireless network testbed which can be dynamically interconnected into specified topologies for wireless network experiments with reproducibility for quantitative evaluation of various new protocols, or application and system concepts [102, 103]. The radio grid emulator as shown in Figure 12 currently consists of 400 wireless nodes having 802.11a/b/g wireless cards laid out in a 20 × 20 grid separated by about 1 m between adjacent nodes. Each node is built on a standard PC platform with multiple wireless and wired network interfaces, some of these nodes can support mobility. The selection of a subset of grid nodes yields a configuration that aims to emulate a wireless network in the real world [102, 103, 104].

**Figure 13.**
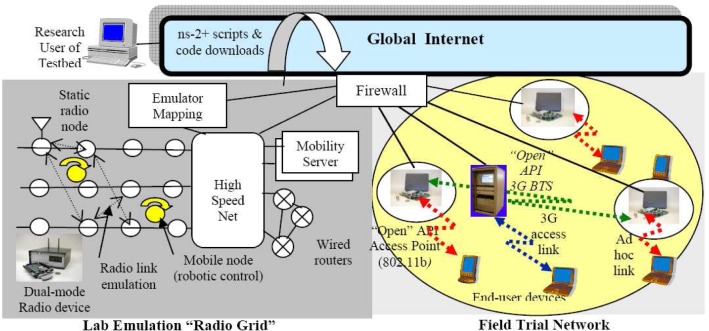
High-level view of proposed 2-tier system architecture for ORBITs.

As shown in Figure 13, the Orbit testbed uses a two-tier architecture with a lab emulator/field trial network architecture to deal with the important issue of reproducibility in experimentation, while at the same time supporting the ability to evaluate protocol and application performance in real-world settings [103]. A user-defined protocol is migrated to the field test after it is validated by the matrix. Users on the Internet can have remote access to the ORBIT [104].

**Figure 14.**
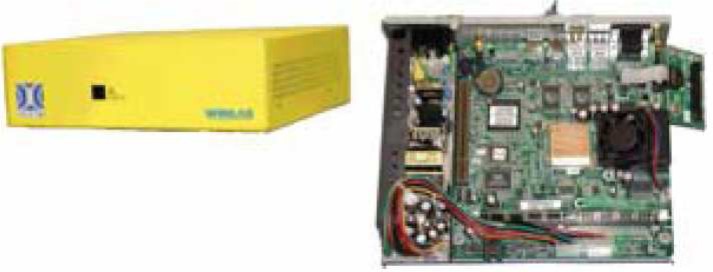
Orbit radio node.

As shown in Figure 14, the radio nodes in ORBIT testbed form the grid and serve as the primary platform for user experiments. Each radio node is a custom wireless node which consists of: (1) 1-GHz VIA C3 processor with 512 MB of RAM and a 20 GB local hard disk, (2) two wireless mini-PCI 802.11a/b/g interfaces, and (3) two 100BaseT Ethernet ports for experimental data and control respectively. The hardware components also include Instrumentation subsystem, Independent WLAN monitor system and support server. These components provide the testbed with power abilities such as radio measurements, MAC/network layer view of the radio grid’s components and huge data storage support [102, 103, 104].

**Figure 15.**
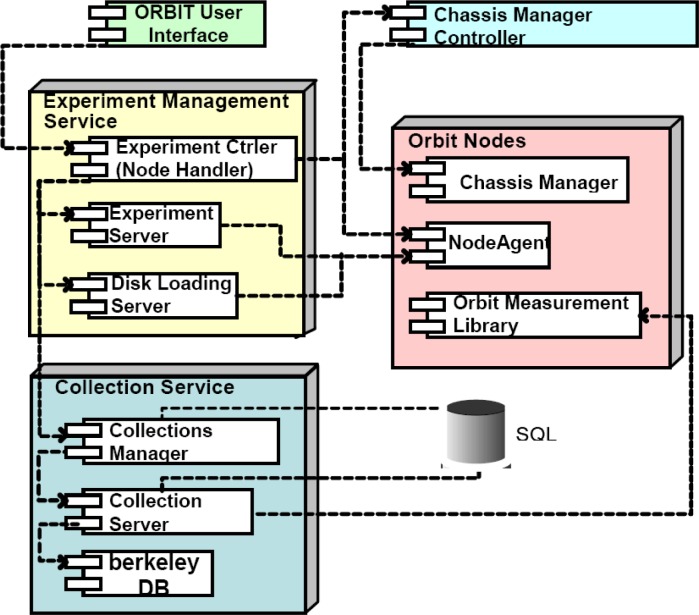
Software architecture of ORBIT testbed.

The Orbit testbed has a software framework as shown in Figure 15, which consists of management/control software as well as user level application. The software packages and libraries support both application/protocol evaluations. These include common libraries for traffic generation, measurement collection, etc., and also provide easy hooks to enable expert users to develop their own applications, protocol stacks, MAC layer modifications and/or other experiments on the testbed. The management/control software include node handler, collection server and disk-loading server. The software for radio nodes include node agent, ORBIT Measurement Library (OML) and Libmac. These components and libraries were developed based on Linux kernel 2.6.4 as the target platform to support the experiment and to provide libraries and interfaces for the user application development [102, 103, 104].

The ORBIT testbed can be used to evaluate various concepts and network applications in real radio-device situations. However, such testbed is primarily applicable to experimentation with higher level networking protocols. The radio node is more like a computer than a real RF radio and does not have the ability to do spectrum sensing experiments. At the same time, the use of only a single vector analyzer limits the exploration of distributed spectrum sensing protocols. Also, the use of standard 100BaseT Ethernet overhead can be a limiting factor in study of channel switching algorithms [94, 103].

### 4.4. WARP platform from Rice Univ

**Figure 16.**
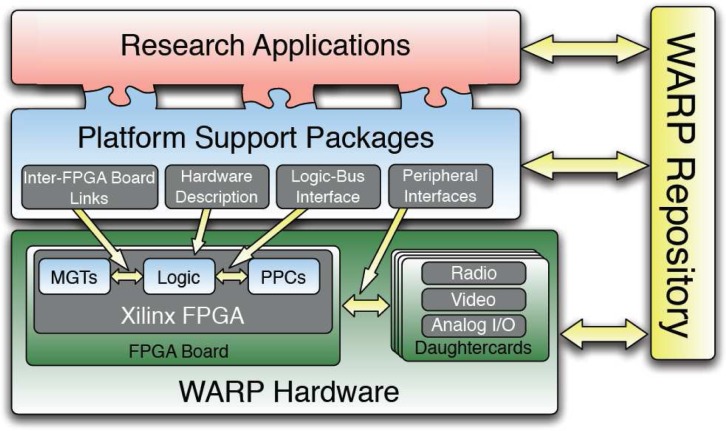
Architecture of the WARP platform.

The WARP (Wireless Open-Access Research Platform) developed by CMC lab at Rice University is a scalable and configurable platform to develop, implement and test advanced wireless algorithms for educational and research oriented applications at both physical layer and MAC layer. The platform architecture, depicted in Figure 16, consists of four key components: custom hardware, platform support packages, open-access repository and research applications; all together providing a reconfigurable wireless testbed [105, 106, 107].

**Figure 17.**
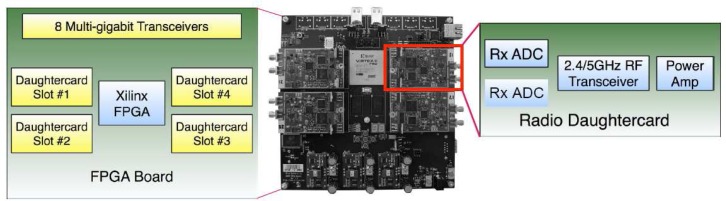
WARP custom hardware, including the Virtex-II Pro FPGA board and radio boards.

The hardware components include a FPGA motherboard and up to four peripheral daughterboards hosted by the FPGA board in its four daughterboard slots as Figure 17 shows. The Xilinx Virtex-II Pro FPGAs is the heart of the hardware and serves as the primary communication processor, the embedded PowerPC core in the Xilinx FPGA was programmed to implement a flexible medium access development framework, which enables researchers to develop network layer designs while abstracting away the physical layer. The four daughterboard slots on the WARP board can be used to build 4 multiple-input multiple-output (MIMO) system [105, 106, 107]. With the radio boards, the testbed may be used for wideband wireless communications in the 2.4 GHz/5 GHz ISM/UNII bands.

Between multiple FPGA boards, the multi-gigabit transceivers (MGTs) built into the Xilinx FPGAs are utilized to provide high speed board-to-board connections which make the WARP platform scalable, each MGT provides a full duplex 3+ Gb/s connection between two FPGAs. The daughterboards can provide analog video capture, playback capabilities, six channels of fast analog I/O (2 A/D and 4 D/A). They can therefore enable the implementation of wireless algorithms in real-time at baseband frequencies, which decouples the processes of algorithmic and RF interface debugging [106].

**Figure 18.**
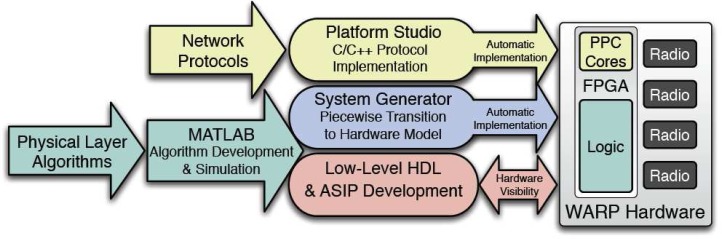
WARP design flows.

For software development, the platform supports different levels of design flows from low level VHDL/Verilog RTL coding to system level MATLAB modeling. Xilinx ISE design tools are used to synthesize hand-coded HDL and map the designs to hardware. Xilinx System Generator, integrated in MATLAB Simulink, provides abstractions for building and debugging high performance DSP systems in MATLAB/Simulink using the Xilinx blockset. Moreover, Simulink hardware co-simulation that expedites the simulation and debugging steps is also supported for MAC and network layer design, the WARP platform supports C language based applications on the PowerPC while interfacing the physical layer implementations in the FPGA fabric [105, 106, 107]. Figure 18 shows how researchers design the various layers of a custom wireless network while using the platform interface tools to integrate different layer implementations.

Rice university also held workshops to further expand the use of the WARP platform at Rice University as well as other universities and research centers. The online open-access repository [108] is the central archive for all source codes, models, platform support packages, application building blocks, research applications, design documents and hardware design files associated with WARP. The researchers can discuss problems and exchange ideas about different algorithmic and hardware implementations.

### 4.5. SFF SDR Platform from Texas Instruments

**Figure 19.**
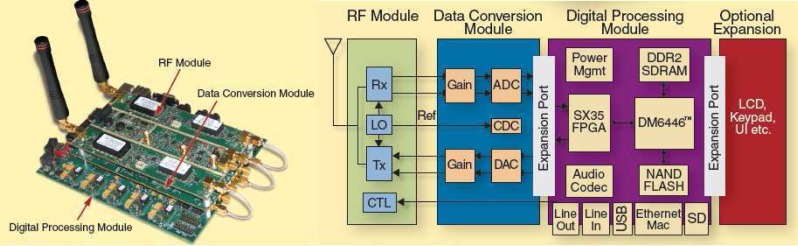
SFF SDR platform and the functional block diagram.

The Small Form Factor (SFF) Software Defined Radio (SDR) development platform provided by Lyrtech in collaboration with Texas Instruments (TI) and Xilinx is a self-contained platform consisting of three separate modules: the digital processing module, the data conversion module and the RF module as shown in Figure 19 [109].

The baseband processing part is designed around the TMS320DM6446 System on Chip (SoC) [110] from TI and Virtex-4 SX35 FPGA from Xilinx. The DM6446 SOC is equipped with a DSP core and a ARM9 general-purpose processor(GPP) core on a single chip, it also comes with a complete set of peripherals necessary for SDR development, including serial, USB and Ethernet ports, as well as DDR2 memory and NAND flash memory [109]. The data conversion module is equipped with a 125 MSPS, 14-bit dual channel ADC and a 500 MSPS 16-bit dual channel interpolating DAC provided by TI. The RF module is configured to have either 5 or 20 MHz bandwidth with working frequencies of 200–930 MHz for the transmitter and 30–928 MHz for the receiver, other higher band products with working frequency from 1.6–2.3 GHz and Wi-Fi band, Wi-Max band are also optional [109]. The platform also uses TI’s MSP430 ultra low-power MCU and power management technology [111].

**Figure 20.**
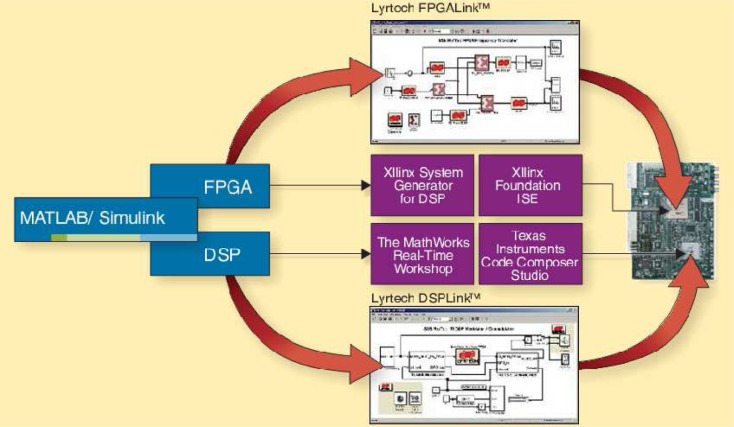
Using SFF SDR platform in module-based design flow.

Lyrtech selected the real-time operating system INTEGRITY from Green Hills Software as the underlying software foundation of the SFF SDR development platforms and integrated various components such as System Generator for DSP from Xilinx, as well as MATLAB, Simulink, and Real-Time Workshop from The MathWorks. These components provide the board’s development package with a module-based design ability. Model-based design supports IP reuse by being able to include legacy code among the other blocks. In the Simulink environment, this is done by using S-functions for the DSP, and black boxes for the FPGA. On the other hand, a developer can integrate his or her model-based algorithms to the low-level coded design of the rest of the team with the use of Embedded Coder, another tool from the Mathworks tailored for embedded processors [109, 111]. The software design flow is shown as Figure 20.

The platform also integrates Software Communications Architecture (SCA) that specifies interactions between hardware and software elements and Common Object Request Broker Architecture (CORBA) communications middleware standard for the SFF SCA Development Platform. This makes the development process much easier [111].

### 4.6. Some TV Band White Space Devices for Cognitive Radio

For years, cognitive radio research has focused on TV band (VHF and UHF spectrum) because this band provides superior propagation and building penetration compared to other unlicensed spectrum in other bands like the 2.4 and 5 GHz bands. After two years white space devices (WSD) testing, the Federal Communications Commission (FCC) in the United States issued a report and order which permits cognitive use of the TV white space spectrum. These new regulatory rules open up an opportunity to develop new wireless networks to utilize this spectrum [112]. The following section will give a brief introduction of the most recent WSD devices from commercial companies such as Motorola, Inc., Adaptrum, Inc. and Philips, Inc. In addition, engineers at the University of Kansas (KU) have built and tested a simulated WSD transmitter and successfully demonstrated how WSD transmissions can be structured to avoid causing harmful interference to licensed broadcasts on adjacent channels.

#### Motorola WSD device

The Motorola WSD platform can operates on channels 21–51 (512 MHz–698 MHz) and includes capabilities for geo-location and sensing of digital TV signals. The system consists of a Cognitive Radio Rack and a laptop computer host connected via Ethernet. The rack consists of a UHF radio and two PRO-3500 carrier boards co-located in a compact Peripheral Component Interconnect (PCI) chassis. The cognitive engine runs on the lower board [113].

This WSD implements a geo-location-based approach as its primary method for the determination of occupied TV channels with a spectrum-sensing capability used to refine the results of the geo-location solution and to prioritize those channels found to be available. This WSD exhibited the fastest scan execution time of 0.1 s/channel [112, 113].

**Figure 21.**
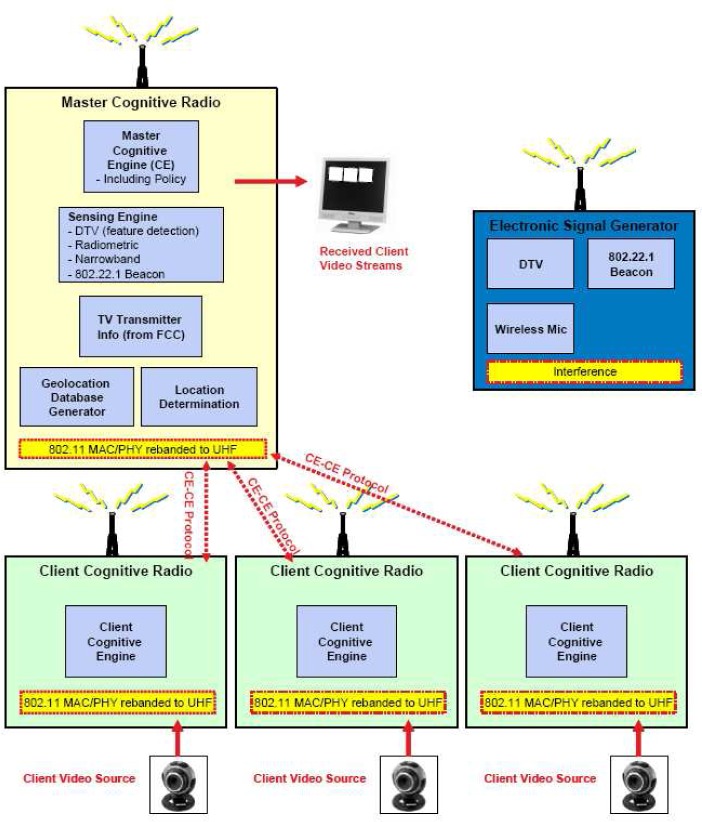
Motorola TV white space cognitive radio demonstration architecture.

In DYSPAN 2008, Motorola WSD platform’s demonstration shows that individual video streams from Client Cognitive radio as transmitter to Master Cognitive radio and displayed on a local terminal. Each of the radios uses a non-proprietary 802.11 MAC/PHY that has been rebanded to UHF. Figure 21 shows the TV white space cognitive radio demonstration architecture.

#### Adaptrum WSD device

Adaptrum Inc.’s Cognitive Radio Platform is an integrated hardware and software development system that has been designed for TV white space operation on UHF television channels 21–51 (512 MHz–698 MHz). The system is capable of various forms of TV signal sensing including wave-form/signature sensing, spectral identification, signal power estimation, and network-level cooperative sensing. It detects both analog and digital TV signals. The system is also capable of signal transmission in the TV bands with flexible waveform, modulation and signal bandwidth construction. It incorporates transmit power control and chain linearization to reduce adjacent channel interference. The maximum transmitter output power specification is 100 mW (+20 dBm) over the selected bandwidth [113, 114].

**Figure 22.**
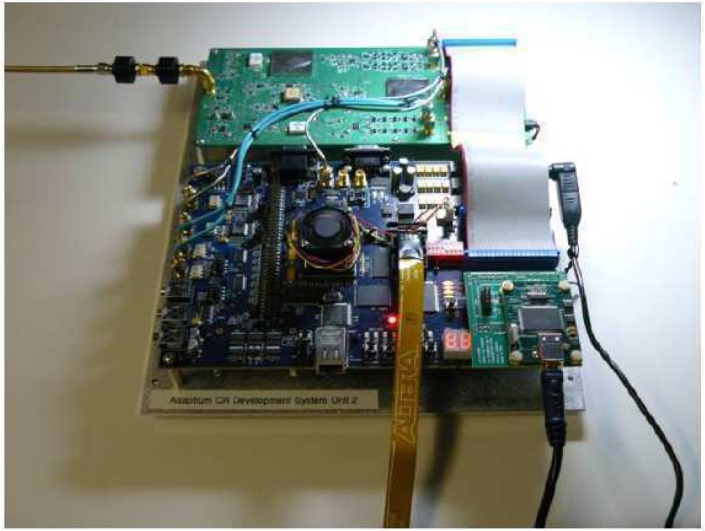
picture of Adaptrum CR prototype system.

Key components of the development platform include a wide-band high dynamic-range RF transceiver operating over the frequency range 400–1000 MHz and an FPGA-based hardware development board with integrated high-speed ADCs and a high-density FPGA where the baseband and protocol–layer functions can be implemented. The software design is based on Matlab-based integrated development environment (IDE) where CR hardware functions are controlled using Matlab GUI and Matlab scripts. Figure 22 shows a lab picture of the prototype system which includes the RF transceiver board and the FPGA board [112, 113, 114].

In the 2008 FCC White Space Device testing, Adaptrum demonstrated its CR prototype system, which is capable of reliably sensing ATSC and NTSC signals at very low detection threshold [112, 114]. More details can be found in [113].

#### Philips WSD device

**Figure 23.**
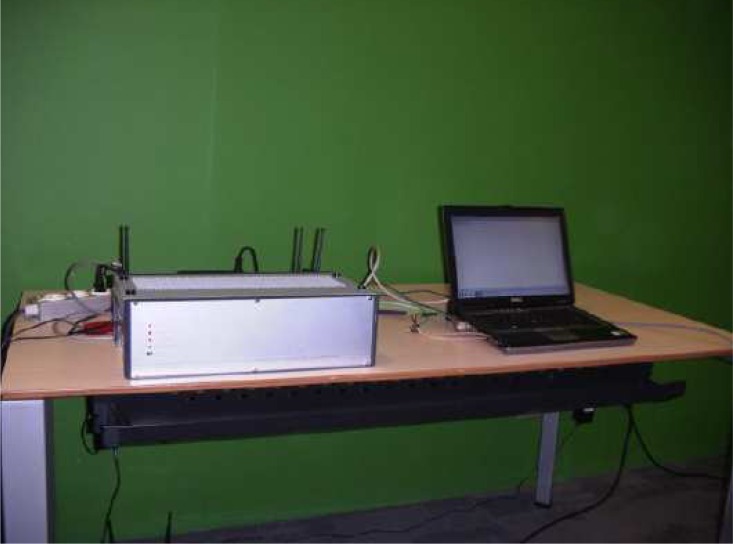
Philips WSD cognitive radio node.

The Philips WSD platform is built using a combination of custom algorithms implemented on a Field Programmable Gate Array (FPGA) and commercial off-the-shelf components. It consists of a commercial TV tuner for tuning to a specified television channel and translating to IF. A digital signal processing board is used for ADC processing and a desktop computer is used to configure the hardware, provide a GUI and store detection results. Figure 23 shows a picture of a cognitive radio node [112, 113].

Philips claims that the prototype WSD will scan UHF channels 21–51 and detect ATSC (DTV), NTSC (analog TV) or wireless microphones to a level of at least −114 dBm over a 6 MHz television channel. The channel scan time for this device varies between 8 and 50 s/ch due to the sequential application of separate ATSC, NTSC and wireless microphone detection algorithms [113].

In the 2008 FCC White Space Device testing, the Philips device demonstrated the most sensitivity in the laboratory tests, also performed best with respect to detecting occupied channels, however, it reported a very high percentage of channels occupied that were potentially available [112, 113].

### 4.7. Other Cognitive Radio Implementation Researches

Other than all the platforms mentioned above, there are many other research centers and universities are involved in cognitive radio implementation research. However, not all of them can be covered in this paper, notable are the platforms from NICT of Japan [115, 116], Shared Spectrum Company [117], Georgia Institute of Technology [118], Virginia Tech University [119] and University of Utah [120].

## 5. Multi-Giga-Hertz Agile Radio Front-End Design

Demand for agile radio is increasing from applications in both communications and radar aspects. A dream agile radio should be able to sense the frequency spectrum, make a best strategy and dynamically access to a desired frequency band. These serial actions must be done as quickly as possible, which poses design and implementation challenges.

In addition to the requirement for quick response, wide frequency range ability is another essential requirement for an agile radio to take full advantage of wide range of spectral availability. A multi-giga-Hertz frequency coverage may sound aggressive, but it is technically achievable, considering recent advances in electronic devices and our experience in UWB radio.

The key subsystem in a multi-giga-Hertz agile radio system is the front-end. The main challenges in designing and implementing such a front-end include: (1) wideband amplifier with low noise figure and large dynamic range, and (2) fast switching between subbands in both transmitter and receiver RF chains.

### 5.1. Potential Front-End Design Options

Scheme 1

Frequency sweeping: like a spectrum analyzer, too slow.

Scheme 2

With multiple narrowbands and working in a hybrid parallel/serial fashion: too many RF analog branches including a filter bank, and band switching takes too much time.

Scheme 3

With a few wide bands and working in a hybrid parallel/serial fashion: less RF analog branches and less band switching time; taking advantage of the power of digital signal process to achieve flexibility and agility.

The scheme 3 seems the most attractive and it is considered in the following.

### 5.2. A Design Example–GigaFront-1 Test-Bed Front-End

Proposed here is a multi-giga-Hertz agile radio front-end design as an optional design for our laboratorial test-bed called GigaFront-1.

Philosophy of Design:

The following aspects are used as guidance in the design.

- cover all frequencies of interest, namely, the busy TV bands and higher bands up to 5.4 GHz
- reduce RF circuit complexity by using large digital processing bandwidth
- achieve fast subband switching in digital domain
- flexible in system configuration and adding new functions/features
- use as many off-the-shelf products as possible

The major frequency parameters are listed in Tables 2 and 3. The proposed transmitter front-end and receiver front-end are shown separately. The overall frequency span ranges from 400 MHz through 5.4 GHz, divided into 10 bands in the transmitter and 6 bands in the receiver. Each band can be further divided into a number of subbands in digital domain. The digital processing bandwidth is 500 MHz, which does not put too much pressure on the data conversion section and digital back-end. Band switching can be done by changing the analog switch positions, combined with local oscillator (LO) frequency switching. In the transmitter, the analog mixer generates upper sidebands and lower sidebands, and depending on the position of the second switch (SW2), one of side bands is utilized. This design assumes a minimum transmit subband bandwidth of 10 MHz, and fast switching between the subbands is achieved by changing the frequencies *f*_0_, *f*_1_ and *f*_2_ in digital domain. Note that *f*_0_, *f*_1_ and *f*_2_ range from −240 MHz to 240 MHz, resulting in a maximum frequency shift 480 MHz. It is expected that switching between the subbands is much faster than switching between the bands. There are three quadrature digital processing cores in parallel in either the transmitter or the receiver. The first digital processing core is dedicated to the two lower busy bands ranging from 400 MHz through 1.4 GHz, while the rest two cores are dedicated to the higher bands with a 4 GHz frequency span. The second and third digital processing cores can work simultaneously to cover a 1 GHz frequency range. In the receiver, the bandwidths are 500 MHz for each of the first two bands and 1 GHz for each of the rest of the four bands. This receiver band arrangement tries to reduce band switching effort in the higher frequency range, assuming unbalanced utilizations in the lower and higher frequency ranges.

### 5.3. Remarks

The multi-giga-Hertz agile radio is a new technical trend in communications and radar applications, in response to the need for efficiently sharing the scarce spectral resource. The front-end design and implementation is the most difficult part in this revolutionary radio. High level front-end design has been proposed through an example. The methodology used here can be applied to different situations, depending on specific frequency band planning, required minimum signal bandwidth, and hardware availability.

## 6. A Compressed Sensing Based Ultra-Wideband Cognitive Radio

Ultra-wideband (UWB) [121, 122, 123, 124] represents a new paradigm in wireless communication. The unprecedented radio bandwidth provides advantages such as immunity from flat fading. However, extremely high sampling rate analog to digital conversion (A/D) becomes a major challenge in UWB communication systems. According to Nyquist sampling theorem, the sampling rate should be at least twice the bandwidth of the signal, and oversampling is required for better quality. For example, a 5 GHz UWB signal needs over 10 Gsps A/D if oversampling is considered, which is not feasible even for the state-of-art hardware.

Compressed Sensing (CS) [21, 125] gives an opportunity to overcome this challenge. The sampling rate can be reduced to less than one tenth of the Nyquist rate, as long as the transmitted signal is sparse in some aspect. CS has been used to UWB communications [126, 127]. A novel CS based UWB communication system is proposed. The channel itself is considered as part of compressed sensing. The hardware complexity of the receiver is moved to the transmitter side. The A/D sampling rate for a 5 GHz UWB signal, covering the 3–8 GHz frequency band, is reduced to as low as 125 Msps [128].

Cognitive Radio (CR) is another challenge in the UWB system. The ultra wide spectrum a UWB system occupied will interfere or be interfered by other narrowband or wideband systems sharing the same spectrum. A simple method is suggested and verified in the CS based UWB communication system.

### 6.1. Compressed Sensing Background

Reference [129] gives a most succinct highlight of the CS principles and will be followed here for a flavor of this elegant theory. Consider the problem of reconstructing an *N* × 1 signal vector *x*. Suppose the basis Ψ = [*ψ*_1_,...,*ψ_N_*] provides a *K*-sparse representation of *x*, where *K* << *N*; that is

(2) $$x=\sum_{n=0}^{N-1}\psi_{n}\theta_{n}=\sum_{l=1}^{K}\psi_{n_{l}}\;\theta_{n_{l}}$$

Here *x* is a linear combination of *K* vector chosen from Ψ; {*n_l_*} are the indices of those vectors; {*θ_n_l__*} are the coefficients. Alternatively, we can write in matrix notation

(3) x=Ψθ

where *θ* = [*θ*_0_,*θ*_1_,..., *θ*_*N*−1_]*^T^*. In CS, *x* can be reconstructed successfully from *M* measurements and *M* << *N*. The measurement vector *y* is done by projecting *x* over another basis Φ which is incoherent with Ψ, i.e., *y* = ΦΨ*θ*. The reconstruction problem becomes an *l*_1_ – *norm* optimization problem:

(4) $$\hat{\theta }=\text{arg}\;\text{min}{\left\|\theta \right\|}_{1}\;\;\;s.t.\;\;\;\;y=\Phi \Psi \theta$$

This problem can be solved by linear programming techniques like basis pursuit (BP) or greedy algorithms such as matching pursuit (MP) and orthogonal matching pursuit (OMP).

When applying the CS theory to communications, the sampling rate can be reduced to sub-Nyquist rate. In [130] and [131] a serial and a parallel system structure were proposed, respectively. Sampling rate can be reduced to less than 20% of the Nyquist rate. However, they were designed for signals that are sparse in frequency domain. In this paper we propose a serial system structure which is suitable for pulse-based UWB communications, which is sparse in time domain. The analog-to-information converter (AIC) structure in [130] is not suitable for UWB communications. The 3–8 GHz UWB signal is considered as an example in describing the reasons:

- The multiplier, which can be a mixer, supporting such high bandwidth for 3–8 GHz UWB signal is difficult to implement.
- The system is time-variant. Each measurement is the product of a streaming signal and a changing PN sequence. This requires a huge amount of storage space and complex computation.

A simple architecture that is suitable for UWB signals is proposed using a finite impulse response (FIR) filter-based architecture.

### 6.2. Filter-based Compressed Sensing

Random filter based CS system for discrete time signals was proposed in [132]. This idea can be extended to continuous time signals. We use * to denote the convolution process in a linear time-invariant (LTI) system. Assume that there is an analog signal *x*(*t*), *t* ∈ [0,*T_x_*] which is *K*-sparse over some basis Ψ:

(5) $$x(t)=\sum_{n=0}^{N-1}\Psi_{n}\;(t)\;\theta_{n}=\Psi \;(t)\;\theta$$

where

(6) $$\Psi \;(t)=\left[\Psi_{0}\;(t),\;\Psi_{1}\;(t),\;\ldots ,\;\Psi_{N-1}\;(t)\right]$$

(7) $$\theta ={\left[\theta_{0},\theta_{1},\ldots ,\theta_{N-1}\right]}^{T}$$

Note that there are only *K* non-zeros in *θ*. *x*(*t*) is then fed into a length-*L* FIR filter *h*(*t*):

(8) $$h\;(t)=\sum_{i=0}^{L-1}h_{i}\;\delta \;\left(t-iT_{h}\right)$$

where *T_h_* is the time delay between each filter tap.

The output *y*(*t*) = *h*(*t*) * *x*(*t*) is then uniformly sampled with sampling period *T_s_*. *T_s_* follows the relation *T_s_*/*T_h_* = *q*, where *q* is a positive integer. *M* samples are collected so that *M*·*T_s_* = ⌊*L*·*T_h_* + *T_x_*⌋, where (*L*·*T_h_* + *T_x_*) is the duration of *y*(*t*).

Now we have the down-sampled output signal *y*(*mT_s_*), *m* = 1,2,...,*M* − 1:

(9) $$\begin{array}{l} y\left({\mathrm{mT}}_{s}\right)=h\left({\mathrm{mT}}_{s}\right)*x\left({\mathrm{mT}}_{s}\right)\\ =\int_{0}^{T_{y}}\;h\left({\mathrm{mT}}_{s}-\tau \right)\;x\;(\tau )\;d\tau \\ =\int_{0}^{T_{y}}\;\left[\sum_{i=0}^{L-1}h_{i}\delta \;\left({\mathrm{mT}}_{s}-{\mathrm{iT}}_{h}-\tau \right)\right]\;x\;(\tau )\;d\tau \\ =\sum_{i=0}^{L-1}h_{i}x\;\left({\mathrm{mT}}_{s}-{\mathrm{iT}}_{h}\right)\\ =\Phi x \end{array}$$

where Φ is a *quasi* – *Toeplitz* matrix and

(10) $$x={\left[x(0),\;x(T_{h}),\;\ldots ,x\left(\left(M-1\right)\;{\mathrm{qT}}_{h}\right)\right]}^{T}=\Psi \theta$$

(11) $$\Psi ={\left[\Psi (0),\;\Psi (T_{h}),\;\ldots ,\;\Psi \left(\left(M-1\right){\mathrm{qT}}_{h}\right)\right]}^{T}$$

A *quasi* – *Toeplitz* matrix has such property: each row of Φ has *L* non-zero entries and each row is a copy of the row above, shifted right by *q* places.

Let *y_m_* = *y*(*mT_s_*), we have

(12) $$y={\left[y_{0},y_{1},\;\ldots ,\;y_{M-1}\right]}^{T}$$

Combining Equations 5, 6, 7, 9, 10, 11 and 12, we have:

(13) y=ΦΨθ=Θθ

Now the problem becomes recovering *N* × 1 vector *θ* from the *M* × 1 measurement vector *y*, which is exactly the same as the problem posed in Equation 4. The number of measurements for successful recovery depends on the sparsity *K*, duration of the analog signal *T_x_*, filter length *L* and the incoherence between Φ and Ψ. Numerical results in Section 6.3. show that when *x*(*t*) is sparse and *h*(*t*) is a PN sequence, *θ* can be reconstructed successfully with a reduced sampling rate, requiring only *M << N* measurements. Note that measurement *y* is a projection from *x* via an FIR filter. We use this feature to design our proposed system.

### 6.3. Compressed Sensing Based UWB Communication System

#### Communication system architecture

**Figure 26.**
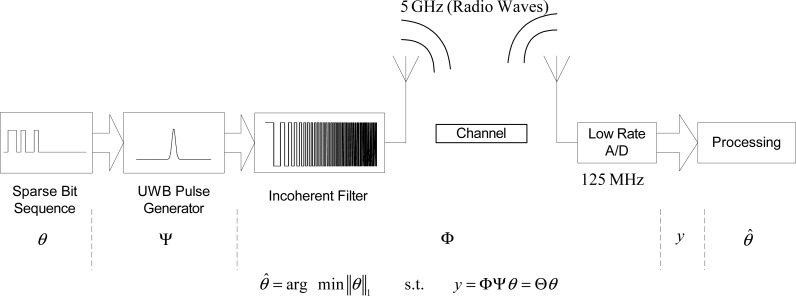
The system architecture of the proposed CS based UWB system. The communication problem of recovering the transmitted information can be modeled as a CS problem.

With the knowledge of Sections 6.1. and 6.2., we propose a CS-based UWB communication system which is able to reduce the sampling rate to 1.25% of the Nyquist rate. The system architecture is illustrated in Figure 26. A UWB signal is transmitted by feeding a sparse bit sequence through a UWB pulse generator and an pre-coding filter. Then, the received signal is directly sampled after the channel, using a low-rate A/D and then processed by a recovery algorithm. Φ is the projection matrix consisting of the pre-coding filter and the channel. It can be noticed that channel itself is part of the projection matrix in CS, so the receiver is very simple, with only one low-rate A/D to collect measurement samples. For example, a 3–8 GHz UWB signals can be successfully recovered by a 125 Msps A/D.

**Figure 27.**
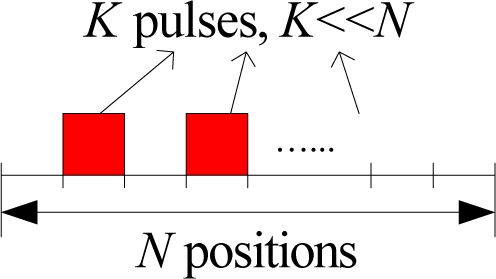
The structure of the *K*-sparse transmitted symbol.

*K*-pulse position modulation (PPM) is used to modulate sparse bit sequence. Each PPM symbol is *K*-sparse: there are *N* positions and only *K << N* pulses in each symbol, as illustrated in Figure 27. The output of the UWB pulse generator can be written using the notations in Equations 5 and 6, with Ψ*_n_*(*t*) = *p*(*t* − *nT_p_*), where *p*(*t*) is the function of the UWB pulse and *T_p_* is the period of the pulse. Pre-coding filter and channel are modeled as FIR filters, with combined impulse response *h*(*t*) = *f*(*t*)*c(*t*), where *f*(*t*) and *c*(*t*) are the impulse response for the pre-coding filter and the channel, respectively. Here *h*(*t*) is equivalent to the *h*(*t*) in Equation 8. The received signal *y*(*t*) = *h*(*t*) * *x*(*t*) is then uniformly sampled by an A/D with sampling period *T_s_*. Similar to Equations 9 and 12, the down-sampled measurements form the *M* × 1 vector *y* = ΦΨ*θ* = Θ*θ*, where Φ is a *quasi* – *Toeplitz* matrix. Now, the communication problem becomes a problem of estimating *θ̂* from *M << N* measurements, which is again identical to the problem described as Equation 4.

The success of recovery relies on the sparsity *K* and the incoherence between Ψ and Φ. Sparsity is easily met by controlling the transmitted sequence. In a simple case, we set *K* = 1, which means that there is only one pulse in PPM symbol. The incoherence property can be met by proper selection of the pre-coding filter *f*(*t*). If *f*(*t*) is a PN sequence whose chip rate is equal to the bandwidth of the UWB pulse *p*(*t*), then *θ* can be successfully recovered using recovery algorithms. So far the discussion is in baseband. If the transmitted UWB is passband, then up-conversion is applied after the pre-coding filter. PN chip rate and the receiver structure remain the same. No down-conversion is required at the receiver. For example, a 3–8 GHz UWB pulse requires a 5 GHz PN chip rate, which is the same as the signal bandwidth, not the Nyquist rate of the maximum signal frequency, as required by the AIC system. A/D at the receiver directly samples the received signal, without doing down-conversion.

The number of measurements *M* and sampling rate are related and determined by the length of the combined filter *h*(*t*). If *h*(*t*) is long, the received signal is “spread out” in the time domain, therefore sufficient measurements can be made under a lower sampling rate.

#### Cognitive radio capability

**Figure 28.**
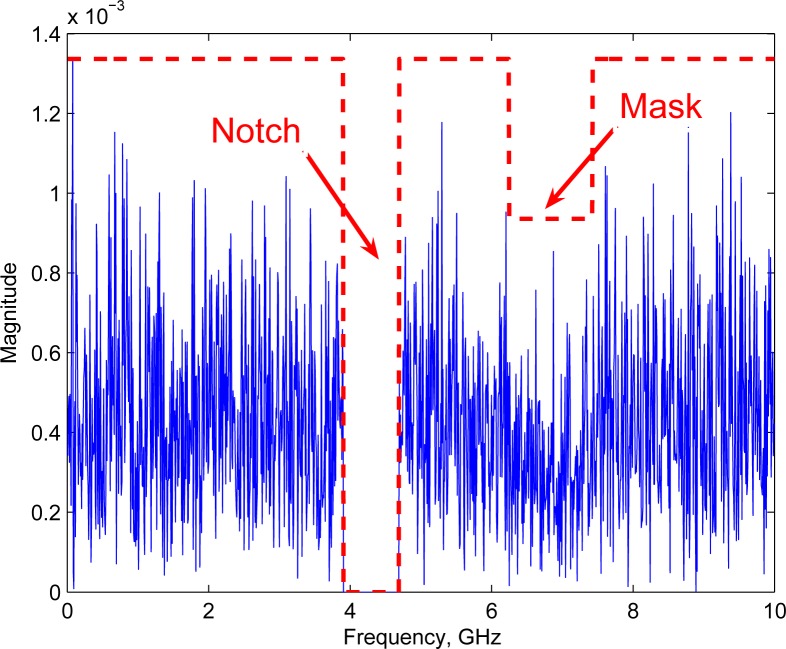
Spectrum Mask of the transmitted signal. The ’notch’ part is set to cancel the interference at the receiver. Transmitter also has the ’notch’ part because CS requires consistency at the receiver and transmitter. The ’mask’ part is set to avoid interfering primary users.

CR concept can be integrated within the CS based architecture in the pre-coding filter, since the spectrum of the transmitted signal is dominated by the spectrum of the pre-coding filter. Suppose the system has the knowledge of the interference frequencies at the receiver and a spectrum mask to avoid interfering other systems. Then, a notch filter will be added at the receiver to cancel the interference. A spectrum mask will be added at the transmitter. From the structure of CS based UWB system, the recover matrix at the receiver should be identical to the pre-coding filter matrix. As a result, the transfer function of the pre-coding filter will notch out some frequencies and set a spectrum mask in a prior manner, as shown in Figure 28. The pre-coding filter is then modified to have the capability to avoid interfering the primary users and canceling the interference from them.

#### Channel estimation

After down-sampling, *y* is processed at the receiver with Θ using BP. In constructing Θ, *f*(*t*), *c*(*t*) and Ψ(*t*) are required. *f*(*t*) and Ψ(*t*) are fixed and can be considered as prior knowledge at the receiver. The channel, *c*(*t*), however, needs to be estimated. A CS based channel estimation method is proposed. A 3–8 GHz channel can be estimated by a 500 Msps A/D.

Similar to Equation 8, the UWB channel can be modeled as:

(14) $$c(t)=\sum_{i=0}^{L-1}c_{i}\delta \;\left(t-{\mathrm{iT}}_{h}\right)$$

**Figure 29.**
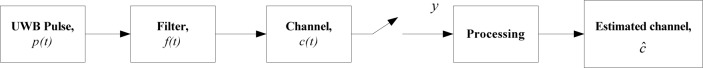
Block diagram of channel estimation.

**Figure 30.**
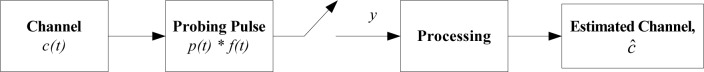
An equivalent block diagram of channel estimation.

The channel estimation block diagram is illustrated in Figure 29. A UWB probing pulse *p*(*t*) * *f*(*t*) is transmitted to “probe” the channel, where *p*(*t*) is a UWB pulse and *f*(*t*) is a PN sequence. At the receiver, sub-Nyquist rate A/D collects *M* uniform measurements. This process can be represented as *y* = *D* ↓ (*c*(*t*) * *f*(*t*) * *p*(*t*)), where *D* ↓ denotes a down-sampling factor of ⌊*N*/*M*⌋ and *y* denotes the measurement vector. Since the system is LTI, an alternative block diagram can be drawn as Figure 30. Then, *y* = *D* ↓ ((*f*(*t*) * *p*(*t*)) * *c*(*t*)). In matrix notation, *y* = Θ*_c_*, where Θ is a *quasi* – *Toeplitz* matrix derived from *f*(*t*) * *p*(*t*) and *c* = [*c*_0_,*c*_1_,...,*c*_*L*−1_]*^T^*. The channel estimation problem is to get *ĉ* from measurements *y*, which is identical to the CS problem described in Equation 4.

Successful recovery requires *c* to be sparse and the incoherent property of measurement matrix Θ [21]. Indoor UWB channel is sparse and PN sequence structured Θ has the incoherent property. PN chip rate should be the same as the bandwidth of the channel under estimation. We demonstrate an estimation result in the following.

**Figure 31.**
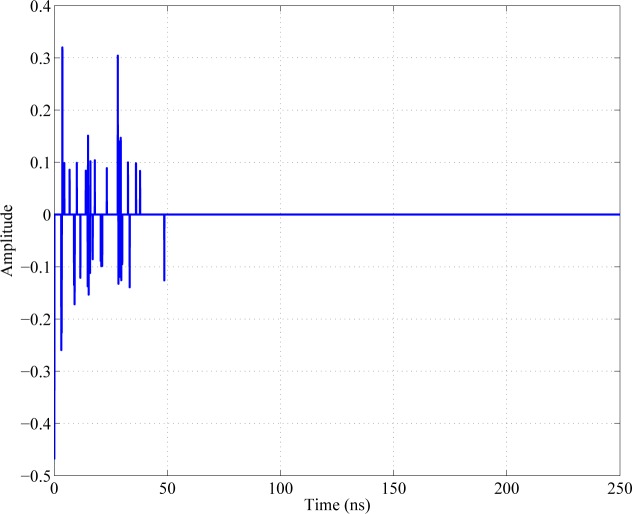
Time domain channel derived from VNA measurement. The sparsity of this channel is 50.

**Figure 32.**
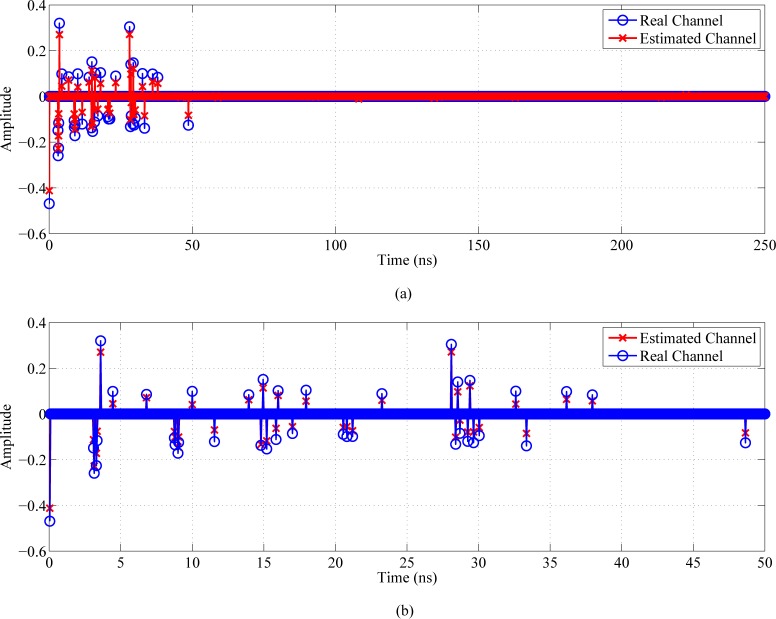
(a) Channel estimation result. (b) Zoomed in version of the result.

First, we need to set up the real channel *c*(*t*) as the estimation target. Vector network analyzer (VNA) is used to get the real indoor channel coefficient *c*. The 3–8 GHz channel is measured by VNA with 1 MHz frequency step and 128 averages. *c*(*t*) (Figure 31) is derived from the VNA data using CLEAN algorithm with a rectangular window. There are about 50 non-zero entries in *c*. PN chip rate is 5 GHz and length of *f*(*t*) is 1 *μs*. Baseband Gaussian UWB pulse *p*(*t*) has 5 GHz bandwidth. Since the measured channel is in passband, up-conversion is applied after the PN filter. At the receiver, 500 Msps A/D is used to get measurements. BP is then used to get the estimated vector *ĉ* with the knowledge of *f*(*t*), *p*(*t*) and *y* only. Additive white Gaussian noise (AWGN) is added at the received samples as *y* = Θ*_c_* + *w*, where *w* is the noise vector. Basis pursuit denoising (BPDN) is used to solve the recovery problem with noise. Figure 32(a) shows the estimation result and Figure 32(b) shows the zoomed in result. It can be seen that although *ĉ* is a little noisy, all major paths in *ĉ* perfectly match to *c*. Only the amplitudes are slightly different.

### 6.4. Discussion

Our proposed approach is to exploit the projection matrix with channel itself and a waveform-based pre-coding at the transmitter. Taking the channel as part of CS results in a very simple receiver design, with only one low-rate A/D. The pre-coding is implemented in a natural way using an FIR filter. The concept has been demonstrated, through simulations, using real-world measurements. Realistic channel estimation is also considered. The philosophy is to trade computation complexity for hardware complexity, and move receiver complexity to the transmitter.

This work is just the beginning of the pre-coded CS. Future work includes reduction of algorithm complexity. Much quicker algorithms are required for real-time applications such as UWB communications.

## 7. Wideband Waveform Optimization for Multiple Input Single Output Cognitive Radio Using Time Reversal

Waveform design or optimization is a key research issue in the current wireless communication system. Waveform should be designed according to the different requirements and objectives of system performance. For example, the waveform should be designed to carry more information to the receiver in terms of capacity. For navigation and geo-location, the ultra short waveform should be used to increase the resolution. If the energy detector is employed at the receiver, the waveform should be optimized such that the energy of the signal in the integration window at the receiver should be maximized. In the context of cognitive radio, waveform design or optimization give us more flexibilities to design radio, which can coexist with other cognitive radios and primary radios. From cognitive radio’s point of view, spectral mask constraint at the transmitter and the influence of Arbitrary Notch Filter at the receiver should be seriously considered for waveform design or optimization, except for the consideration of the traditional communication objectives. Spectral mask constraint is imposed on the transmitted waveform such that cognitive radio has no interference to primary radio, whereas Arbitrary Notch Filter at the receiver is used to cancel the interference from primary radio to cognitive radio.

**Figure 33.**
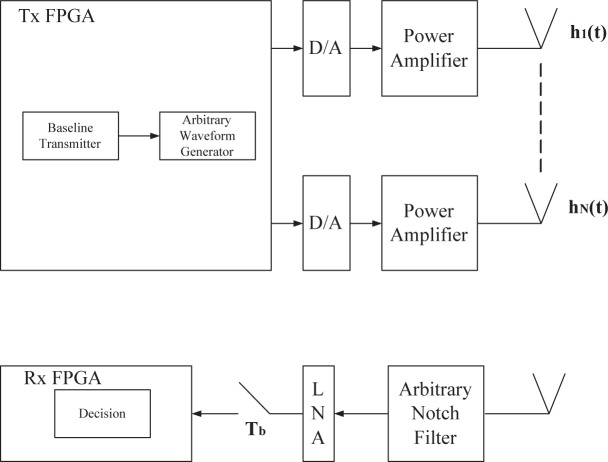
System architecture.

This section deals with wideband waveform optimization for multiple input single output (MISO) cognitive radio using time reversal. The system architecture is shown in Figure 33. We limit our discussion to a single user scenario. There are *N* antennas at the transmitter and one antenna at the receiver. OOK modulation is used for transmission. Thus the transmitted signal at the transmitter antenna *n* is,

(15) $$s_{n}\;(t)=\sum_{j=-\infty }^{\infty }d_{j}p_{n}\;\left(t-{\mathrm{jT}}_{b}\right)$$

where *T_b_* is the bit duration, *p_n_*(*t*) is the transmitted bit waveform defined over [0,*T_p_*] at the transmitter antenna *n* and *d_j_* ∈ {0, 1} is *j*-th transmitted bit. Without loss of generality, the minimal propagation delay is assumed to be zero. The energy of transmitted waveforms is *E_p_*,

(16) $$\sum_{n=1}^{N}\int_{0}^{T_{p}}p_{n}^{2}\;(t)\;\mathrm{df}=E_{p}$$

The received noise-polluted signal at the output of low noise amplifier (LNA) is,

(17) $$r(t)=\sum_{n=1}^{N}h_{n}\;(t)⊗s_{n}\;(t)+n\;(t)$$

(18) $$=\sum_{j=-\infty }^{\infty }d_{j}\sum_{n=1}^{N}x_{n}\;\left(t-{\mathrm{jT}}_{b}\right)+n\;(t)$$

where *h_n_*(*t*), *t* ∈ [0,*T_h_*] is the multipath impulse response that takes into account the effect of channel impulse response, the RF front-ends in the transceivers such as Power Amplifier, LNA and Arbitrary Notch Filter as well as antennas between the transmitter antenna *n* and the receiver antenna. *h_n_*(*t*) is available at the transmitter. $\int_{0}^{T_{h}}\;h_{n}^{2}\;(t)\;\mathrm{dt}=E_{\mathrm{nh}}$. “⊗” denotes convolution operation. *n*(*t*) is AWGN. *x_n_*(*t*) is the received noiseless bit-“1” waveform defined as

(19) $$x_{n}(t)=h_{n}(t)⊗p_{n}(t)$$

We further assume that $T_{b}\geq T_{h}+T_{p}\overset{\text{def}}{=}T_{x}$, i.e., no existence of ISI.

If the waveforms at different transmitter antennas are assumed to be synchronized, the *k*-th decision statistic is,

(20) $$r\left({\mathrm{kT}}_{b}+t_{0}\right)=\sum_{j=-\infty }^{\infty }d_{j}\sum_{n=1}^{N}x_{n}\;\left({\mathrm{kT}}_{b}+t_{0}-{\mathrm{jT}}_{b}\right)+n\;(t)$$

(21) $$=d_{k}\sum_{n=1}^{N}x_{n}\;(t_{0})+n\;(t)$$

In order to maximize the system performance, $\sum_{n=1}^{N}x_{n}\;(t_{0})$ should be maximized. Thus the optimization problem can be formulated as follows to get the optimal waveforms *p_n_*(*t*),

(22) $$\begin{array}{l} \text{max}\;\sum_{n=1}^{N}x_{n}\;(t_{0})\\ s.t.\;\sum_{n=1}^{N}\;\int_{0}^{T_{p}}\;p_{n}^{2}\;(t)\;\mathrm{df}\;\leq \;E_{p}\\ \;\;\;\;\;0\leq t_{0}\leq T_{b} \end{array}$$

An iterative algorithm is proposed here to give the optimal solution to the optimization problem, which is a computationally efficient algorithm. For the simplicity of the following presentation, *t*_0_ is assumed to be zero. Meanwhile,

(23) $$x\;(t)=\sum_{n=1}^{N}x_{n}\;(t)$$

From inverse Fourier transform,

(24) $$x_{\mathrm{nf}}\;(f)=h_{\mathrm{nf}}\;(f)\;p_{\mathrm{nf}}\;(f)$$

and

(25) $$x_{f}\;(f)=\sum_{n=1}^{N}h_{\mathrm{nf}}\;(f)\;p_{\mathrm{nf}}\;(f)$$

where *x_nf_* (*f*), *h_nf_* (*f*) and *p_nf_* (*f*) are the frequency domain representations of *x_n_*(*t*), *h_n_*(*t*) and *p_n_*(*t*) respectively. *x_f_* (*f*) is frequency domain representation of *x*(*t*). Thus,

(26) $$x\;(0)=\sum_{n=1}^{N}x_{n}\;(0)$$

and

(27) $$x_{n}\;(0)=\int_{-\infty }^{\infty }\;x_{\mathrm{nf}}\;(f)\;\mathrm{df}$$

If there is no spectral mask constraint, then according to the Cauchy–Schwarz inequality,

(28) $$x\;(0)=\sum_{n=1}^{N}\;\int_{-\infty }^{\infty }\;h_{\mathrm{nf}}\;(f)\;p_{\mathrm{nf}}\;(f)\;\mathrm{df}$$

(29) $$\leq \sum_{n=1}^{N}\;\sqrt{\int_{-\infty }^{\infty }\;{\left|h_{\mathrm{nf}}\;(f)\right|}^{2}\mathrm{df}\;\int_{-\infty }^{\infty }\;{\left|p_{\mathrm{nf}}\;(f)\right|}^{2}\mathrm{df}}$$

(30) $$\leq \sqrt{\sum_{n=1}^{N}\;\int_{-\infty }^{\infty }\;{\left|h_{\mathrm{nf}}\;(f)\right|}^{2}\mathrm{df}}\;\;\sqrt{\sum_{n=1}^{N}\;\int_{-\infty }^{\infty }\;{\left|p_{\mathrm{nf}}\;(f)\right|}^{2}\mathrm{df}}$$

(31) $$=\sqrt{E_{p}\sum_{n=1}^{N}\;E_{\mathrm{nh}}}$$

when *p_nf_* (*f*) = *αh_nf_* (*f*) for all *f* and *n*, two equalities are obtained.

(32) $$\alpha =\sqrt{\frac{E_{p}}{\sum_{n=1}^{N}\;\int_{-\infty }^{\infty }\;{\left|h_{\mathrm{nf}}\;(f)\right|}^{2}\mathrm{df}}}$$

In this case, *p_n_* (*t*) = *αh_n_* (−*t*), which means the optimal waveform *p_n_*(*t*) is the corresponding time reversed multipath impulse response *h_n_*(*t*).

If there is spectral mask constraint, then the following optimization problem will become more complicated,

(33) $$\begin{array}{l} \text{max}\;x\;(0)\\ s.t.\;\sum_{n=1}^{N}\;\int_{0}^{T_{p}}\;p_{n}^{2}\;(t)\;\mathrm{df}\leq E_{p}\\ \;\;\;\;\;{\left|p_{\mathrm{nf}}\;(f)\right|}^{2}\leq c_{\mathrm{nf}}\;(f) \end{array}$$

where *c_nf_*(*f*) represents the arbitrary spectral mask constraint at the transmitter antenna *n*.

Because *p_nf_*(*f*) is the complex value, the phase and the modulus of *p_nf_*(*f*) should be determined.

Meanwhile,

(34) $$x\;(0)=\int_{-\infty }^{\infty }\;x_{f}\;(f)\;\mathrm{df}$$

and

(35) $$x_{f}\;(f)=\sum_{n=1}^{N}\left|h_{\mathrm{nf}}\;(f)\right|\;\left|p_{\mathrm{nf}}\;(f)\right|\;e^{j2\pi \left(\text{arg}\left(h_{\mathrm{nf}}\;(f)\right)+\text{arg}\left(p_{\mathrm{nf}}\;(f)\right)\right)}$$

where the angular component of the complex value is arg (•).

For the real value signal *x*(*t*),

(36) $$x_{f}\;(f)=x_{f}^{*}\;(-f)$$

where “*” denotes conjugate operation. Thus,

(37) $$x_{f}\;(-f)=\sum_{n=1}^{N}\left|h_{\mathrm{nf}}\;(f)\right|\;\left|p_{\mathrm{nf}}\;(f)\right|\;e^{-j2\pi \left(\text{arg}\left(h_{\mathrm{nf}}\;(f)\right)+\text{arg}\left(p_{\mathrm{nf}}\;(f)\right)\right)}$$

and *x_f_*(*f*) + *x_f_*(−*f*) is equal to

(38) $$\sum_{n=1}^{N}\left|h_{\mathrm{nf}}\;(f)\right|\;\left|p_{\mathrm{nf}}\;(f)\right|\;\text{cos}\left(2\pi \;\left(\text{arg}\left(h_{\mathrm{nf}}\;(f)\right)+\text{arg}\left(p_{\mathrm{nf}}\;(f)\right)\right)\right)$$

If *h_nf_*(*f*) and |*p_nf_* (*f*)| are given for all *f* and *n*, maximization *x*(0) is equivalent to setting,

(39) $$\text{arg}\;\left(h_{\mathrm{nf}}\;(f)\right)+\text{arg}\;\left(p_{\mathrm{nf}}\;(f)\right)=0$$

which means the angular component of *p_nf_*(*f*) is the negative angular component of *h_nf_*(*f*).

The optimization problem (33) can be simplified as,

(40) $$\begin{array}{l} \text{max}\;\sum_{n=1}^{N}\;\int_{-\infty }^{\infty }\;\left|h_{\mathrm{nf}}\;(f)\right|\;\left|p_{\mathrm{nf}}\;(f)\right|\;\mathrm{df}\\ s.t.\;\sum_{n=1}^{N}\;\int_{-\infty }^{\infty }\;{\left|p_{\mathrm{nf}}\;(f)\right|}^{2}\;\mathrm{df}\leq E_{p}\\ \;\;\;\;\;{\left|p_{\mathrm{nf}}\;(f)\right|}^{2}\leq c_{\mathrm{nf}}\;(f) \end{array}$$

Because

(41) $$\left|h_{\mathrm{nf}}\;(f)\right|=\left|h_{\mathrm{nf}}\;(-f)\right|$$

(42) $$\left|p_{\mathrm{nf}}\;(f)\right|=\left|p_{\mathrm{nf}}\;(-f)\right|$$

(43) $$\left|c_{\mathrm{nf}}\;(f)\right|=\left|c_{\mathrm{nf}}\;(-f)\right|$$

for all *f* and *n*. Thus uniformly discrete frequency points *f*_0_, ..., *f_M_* are considered in the optimization problem (40). Meanwhile, *f*_0_ corresponds to the DC component and *f*_1_, ..., *f_M_* correspond to the positive frequency components.

Define column vectors h*_f_*, h_1*f*_, ..., h*_Nf_*,

(44) $$h_{f}={\left[h_{1f}^{T}\;\;h_{2f}^{T}\;\cdots \;h_{Nf}^{T}\right]}^{T}$$

(45) $${\left(h_{\mathrm{nf}}\right)}_{i}=\{\begin{array}{c} \left|h_{\mathrm{nf}}\;\left(f_{i-1}\right)\right|,\;i=1\\ \sqrt{2}\left|h_{\mathrm{nf}}\;\left(f_{i-1}\right)\right|,\;i=2,\ldots ,M+1 \end{array}$$

where “*T*” denotes transpose operation.

Define column vectors p*_f_*, p_1*f*_, ..., p*_Nf_*,

(46) $$p_{f}={\left[p_{1f}^{T}\;\;\;p_{2f}^{T}\;\cdots \;p_{Nf}^{T}\right]}^{T}$$

(47) $${\left(p_{\mathrm{nf}}\right)}_{i}=\{\begin{array}{c} \left|p_{\mathrm{nf}}\;\left(f_{i-1}\right)\right|,\;i=1\\ \sqrt{2}\left|p_{\mathrm{nf}}\;\left(f_{i-1}\right)\right|,\;i=2,\ldots ,M+1 \end{array}$$

Define column vectors c*_f_*, c_1*f*_, ..., c*_Nf_*,

(48) $$c_{f}={\left[c_{1f}^{T}\;\;c_{2f}^{T}\;\cdots \;c_{Nf}^{T}\right]}^{T}$$

(49) $${\left(c_{\mathrm{nf}}\right)}_{i}=\{\begin{array}{c} \sqrt{\left|c_{\mathrm{nf}}\;\left(f_{i-1}\right)\right|},\;i=1\\ \sqrt{2\left|c_{\mathrm{nf}}\;\left(f_{i-1}\right)\right|},\;i=2,\ldots ,M+1 \end{array}$$

Thus, the discrete version of the optimization problem (40) is shown below,

(50) $$\begin{array}{l} \text{max}\;h_{f}^{T}\;p_{f}\\ s.t.\;{\left\|p_{f}\right\|}_{2}^{2}\leq E_{p}\\ \;\;\;\;\;0\leq p_{f}\leq c_{f} \end{array}$$

An iterative algorithm is shown as follows.

1. Initialization: *P* = *E_p_* and $p_{f}^{*}$ is set to be all-0 column vector.
2. Solve the following optimization problem to get the optimal $q_{f}^{*}$ using Cauchy–Schwarz inequality.
  (51) $$\begin{array}{l} \text{max}\;h_{f}^{T}\;q_{f}\\ s.t.\;{\left\|q_{f}\right\|}_{2}^{2}\leq P \end{array}$$
3. Find *i* such that ${\left(q_{f}^{*}\right)}_{i}$ is the maximal value in the set $\left\{{\left(q_{f}^{*}\right)}_{j}|{\left(q_{f}^{*}\right)}_{j}>{\left(c_{f}\right)}_{j}\right\}$. If {*i*} = ∅, then the algorithm is terminated and $p_{f}^{*}:=p_{f}^{*}+q_{f}^{*}$. Otherwise go to step 4.
4. Set ${\left(p_{f}^{*}\right)}_{i}={\left(c_{f}\right)}_{i}$.
5. $P:=P-{\left(c_{f}\right)}_{i}^{2}$ and set (h*_f_*)*_i_* to zero. Go to step 2.

When $p_{f}^{*}$ is obtained for the optimization problem (50), the optimal *p_nf_*(*f*) and the corresponding *p_n_*(*t*) can be smoothly achieved.

## 8. A Unified Framework for Cognitive Radio and Cognitive Radar

### 8.1. A Unified Framework for Cognitive Radio and Cognitive Radar

**Figure 34.**
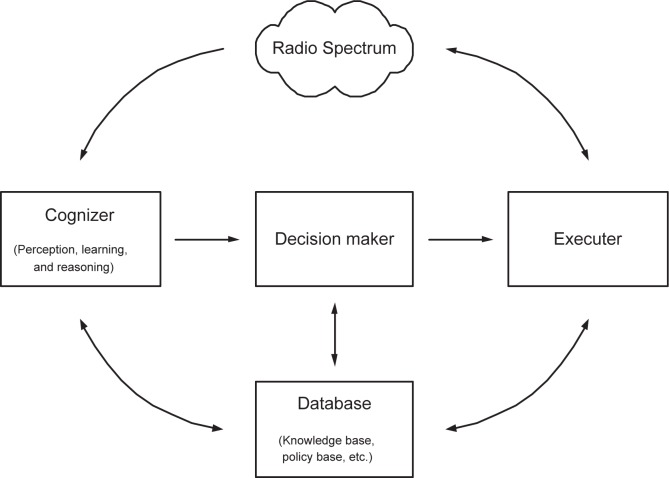
A unified framework for cognitive radio and cognitive radar.

Cognitive Radio (CR) evolves from Software Defined Radio (SDR) and it introduces intelligence to radio systems. One of the key features of cognitive radio is the capability of learning. A framework for cognitive radio is shown in Figure 34, which includes four units: cognizer, decision maker, executer, and database.

A cognizer has the capability of perceiving the radio spectrum. Moreover, it can learn and even reason from what it perceives. The spectrum sensing of cognitive radio, which senses the availability of certain frequency segments of the radio spectrum with certain time slot, is included in this unit. Some mathematical tools for the cognizer can be borrowed from other disciplines, such as machine learning and aritficial intellegence [133, 134, 135]. At the perception phase, the spectrum is perceived with certain time slot and the perceived signals are further processed (including transformations and modeling). Furthermore, feature parameters can be extracted. At the learning and reasoning phase, the processed perceived signals or the extracted feature parameters are used for learning the spectrum (e.g., by training) and further reasoning (such as predicting the status of channel and recognizing the extracted feature parameters). In a word, the cognizer perceives the radio spectrum, and learns and reasons from it. The cognizer outputs reference information of the radio spectrum to decision maker. The following mathematical tools can work for the cognizer.

#### Hidden Markov Model

Hidden Markov Model (HMM) is a widely-used statistics model for sequential data. It maps observations to hidden states with probabilities and supports transitions of hidden states. Basically, it deals with three kinds of problems. One is called learning (or training) which is the generation of HMM parameters using one or more sequences of observations. The second kind of problem is to find the probability of a sequence of observation with given HMM parameters. The third one is decoding, i.e., finding the sequence of hidden states with a given sequence of observations. Our work on spectrum recognition using HMM can be found in [136].

#### Bayesian Network

Bayesian Network (BN) is a graphic model which explicitly uncovers the probabilistic structure of dependency in a set of random variables. It uses a direct acyclic graph (DAG) to represent the dependency structure, in which each node denotes a random variable and each edge denotes the relation of dependency. The key difference between BN and HMM is that the former represents the hidden states using a set of random variables instead of a single random variable [137, 138, 139]. BN is a static model. While Dynamic Bayesian Network (DBN) is a powerful tool to model the sequential data or the dynamic system. DBN can be employed in the context of cognitive radio or cognitive radar to model the spectrum. For modeling the spectrum, the main task is learning, which means statistic information is extracted from the measured training data and DBN is built. There are two stages of learning. One is structure learning, i.e., topology selection. The other is parameter learning, i.e., parameter estimation. In the stage of structure learning, we need to determine the topology of DBN, i.e., the structure of dependency. In the stage of parameter learning, the conditional probability distribution (CPD) of each node should be estimated. After DBN is built, we can use it to do filtering, prediction, classification and so on, all of which can be called inference. In our work, DBN is exploited to predict the state of the specific spectrum. The results of prediction will be conveyed to decision maker and decision maker will make the control decision for the behavior of cognitive radio or cognitive radar.

#### Logistic Regression

In contrast to BN, which models the dependency explicitly, logistic regression models the dependency in an implicit and linear manner and provides a direct prediction of spectrum activity. The advantage of logistic regression is that it is simple and can give the probability of prediction. Mathematically, logistic regression can be written as,

(52) $$\text{log}\;\left(\frac{p\;(S_{i}=1)}{p\;(S_{i}=0)}\right)=\sum_{k=1}^{N}\beta_{k}\;f\left(S_{i-k}\right)$$

where *β_k_s* are regression coefficients to be estimated from training data. *f* is a function of state.

The database in the proposed framework provides a storage for knowledge, policy and other data. The decision maker in Figure 34 chooses a policy for execution based on the information provided by the cognizer and the knowledge from the database. Partially Observable Markov Decision Process (POMDP) can be used for the decision process [140]. POMDP models the interaction procedure of an agent with outside world. The solution of POMDP is the optimal policy for choosing actions. Solving a POMDP is not easy. The first detailed algorithms for finding exact solutions of POMDP were introduced in [141]. There exists some software tools for solving POMDPs, such as pomdp-solve [142], MADP [143], ZMDP [144], APPL [145], and Perseus [146]. Among them, APPL is the fastest one in most cases [145].

The idea of cognitive radar was put forward in [147]. The framework shown in Figure 34 can also be applied to cognitive radar. The major difference between cognitive radio and cognitive radar is the implementation of executer. For cognitive radio, a traditional wireless communication device or SDR can be used as the executer. While for cognitive radar, the executor can be the current radar system.

### 8.2. Measurements of Wideband Time-domain Signals

**Figure 35.**
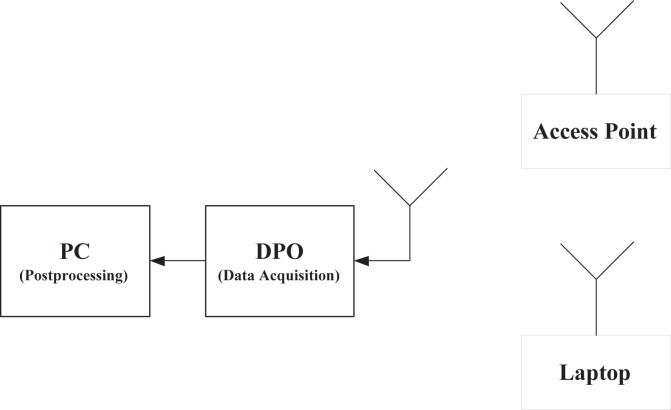
Setup of the measurement.

**Figure 36.**
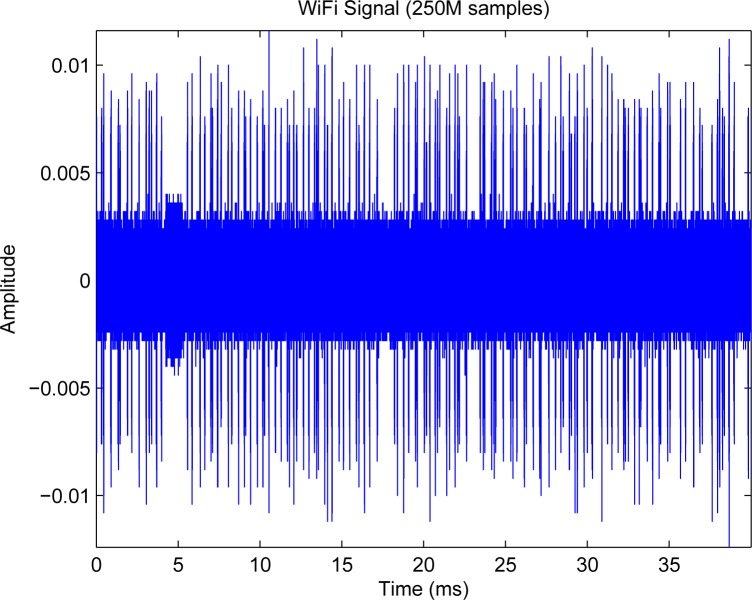
Measured wideband time-domain signals.

**Figure 37.**
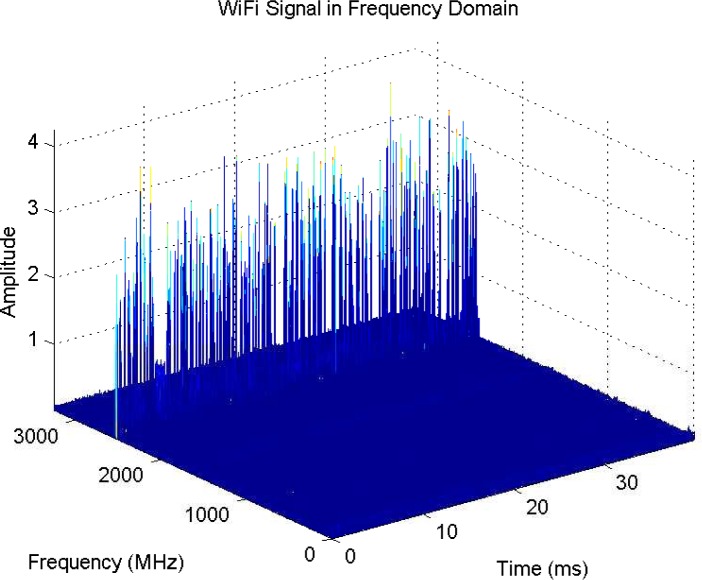
Time-frequency graph of measured sigals.

Wideband time-domain signals were measured in Tennessee Technological University using Digital Phosphor Oscilloscope (DPO). The model of DPO that we used is Tektronix DPO72004, which supports a maximum bandwidth of 20 GHz and a maximum sampling rate of 50 GS/s. Figure 35 depicts the setup of the measurement. In the measurement, a laptop accessed the internet through a wireless Wi-Fi router. An antenna whose frequency range is 800–2500 MHz was placed near the laptop and connected to DPO. The measured time-domain signals are shown in Figure 36. Fast Fourier Transform (FFT) was applied to the measured signals and the resulting time-frequency graph is shown in Figure 37.

The measured data were used to evaluate our prediction algorithms for spectrum sensing in cognitive radio. One prediction algorithm is based on HMM. The other one is based on Logic Regression [148]. The research of BN based prediction is currently underway.

## 9. Quickest Spectrum Sensing

### 9.1. Single Channel and Non-Bayesian Case

In this subsection, we consider the case of monitoring a single frequency channel in which the detector has no *a priori* information about the emergence time of primary user. For simplicity, we assume that, at the beginning, there is no primary user and the primary user could emerge at any time *T* (it is also possible that it never emerges, then *T* = ∞). We denote by *X_t_* the *t*-th observation. Meanwhile, the probability density functions (we assume that they exist) of observation when primary user exists or not are denoted by *p*_1_ and *p*_0_, respectively.

#### Performance Metrics

**Figure 38.**
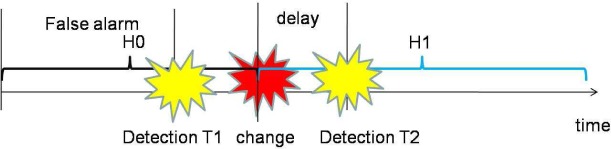
Illustration of detection delay and false alarm.

We denote by T* the time that the secondary user claims that the primary user emerges. As illustrated in Figure 38, when T* < *T*, false alarm happens (e.g., *T*_1_ in Figure 38); and when T* > *T*, there is a detection delay (e.g., *T*_2_ in Figure 38). Both incur performance penalties. Then, we define the following two performance metrics:

- Detection delay average run length (ARL) ((*x*)^+^ equals *x* when *x* > 0 and 0 otherwise):
  (53) $$d≜E\left[{\left(T*-T\right)}^{+}\right]$$
- False alarm ARL
  (54) f≜E[T*|T=∞]

#### CUSUM Test

A popular approach for detecting the change is cumulative sum (CUSUM) test, originally proposed by Page in 1954 [78, 149]. The asymptotic optimality of CUSUM test was proved by Lorden in 1971 [150]. As a more difficult problem, the non-asymptotic optimality of CUSUM test was proved by Moustakides in 1986 [151]. In CUSUM test, the test statistic, denoted by *m_t_* at time slot *t*, is given by

(55) $$m_{t}=\text{max}\left(0,\;m_{t-1}+L(t)\right)$$

where *L*(*t*) is the log likelihood of observation received at time slot *t*, which is given by

(56) $$L\left(t\right)=\text{log}\;\frac{P_{1}\left(X_{t}\right)}{P_{0}\left(X_{t}\right)}$$

Intuitively, the test statistic *m_t_* is the sum of log likelihood bounced by the boundary *m_t_* = 0. Obviously, the larger *m_t_* is, the more probably the change has happened (since the distribution is more biased to that after change). Then, the random stopping time of claiming the change is given by

(57) $$s=\text{min}\left\{t|m_{t}\geq \gamma \right\}$$

where *γ* is a predetermined threshold.

Another equivalent form of CUSUM test is to set a random walk for every time slot: for time *t*, we define random walk

(58) $$q_{t}\;(\tau )=\sum_{n=t}^{\tau }L(n)$$

and stopping time

(59) $$s_{t}=\text{min}\;\left\{\tau |q_{t}(\tau )\geq \gamma \right\}$$

where the threshold *γ* is the same as that in (57). From the family of stopping times {*s_t_*}_*t*=1,2,..._, we choose the earliest one as the time claiming the change of distribution, i.e.,

(60) $$s^{\prime }=\text{min}\left\{s_{t},\;t=1,2,\ldots \right\}$$

It is easy to verify that *s* = *s*′. Therefore, both approaches are equivalent. In contrast to the former approach (we call it *single metric* approach), the latter (we call it *multiple random walk* approach) requires infinite memory (for each time slot, we need some memory to store the updated random walk value). Therefore, the former is more suitable for practical systems. However, the latter approach can provide some hints to approximate algorithms, as we will see.

#### Quickest Detection with Unknown Parameters

In the standard CUSUM test, it is assumed that the distributions before and after the change are perfectly known to the detector. Unfortunately, in many situations, the distribution after the change in cognitive radio systems is not completely known. For example, if we use received power as the observation, the exact value of average received power after the change is unknown although we know that the average received power is increased.

Let us take the detection of pilot in digital TV (DTV) systems for instance. On ignoring the interference leaked from signals in neighboring spectrum, the expressions of received signal are given by

(61) $$\{\begin{array}{ll} H_{0}: & r(t)=n(t)\\ H_{1}: & r(t)=A\;\text{sin}\;\left(\omega tT_{O}+\phi \right)+n(t) \end{array}$$

where *n*(*t*) is additive white Gaussian noise (AWGN) with zero mean and variance $\sigma_{n}^{2}$ (we assume that $\sigma_{n}^{2}$ is known to the detector), *A* is the received amplitude of pilot, *ω* is the angular frequency of pilot, *T_O_* is the time interval between two consecutive observations and *ϕ* is the phase. Note that *ω* can be found from the DTV system specification while both *A* and *ϕ* are unknown. Therefore, we cannot apply the CUSUM test directly.

One approach to tackle the unknown parameter is to apply the generalized likelihood ratio (GLR), in which the unknown parameters are replaced with their maximum likelihood estimation. Another alternative is to adopt the philosophy of multiple random walk approach of CUSUM test [75]. In such an approach, called parallel CUSUM test, we set a family of (say, *N*) parameter candidates {(*A_n_*,*ϕ_n_*)}_*n*=1,...,*N*_. We do CUSUM test for each parameter candidate by assuming that the candidate is the true value of the parameter. Then, we obtain a family of stopping times {*s_n_*}_*n*=1,2,...,*N*_. The stopping time of the parallel CUSUM test is obtained by choosing the earliest stopping time, i.e.,

(62) $$s*=\text{min}\;\left\{s_{n},\;n=1,2,\ldots ,N\right\}$$

**Figure 39.**
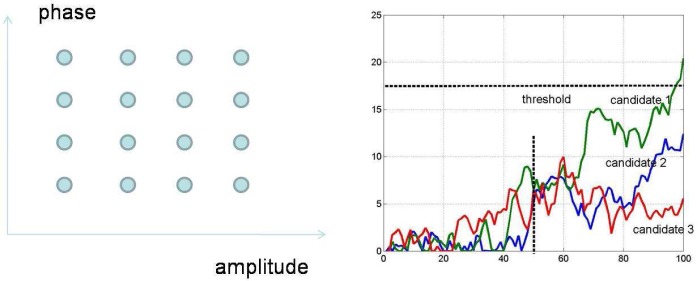
Illustration of parallel CUSUM test.

The procedure of parallel CUSUM test is illustrated in Figure 39. The left part shows a possible selection of parameter candidates (we choose a grid in the product space of amplitude and phase). The right part shows the competition of several parameter candidates.

**Figure 40.**
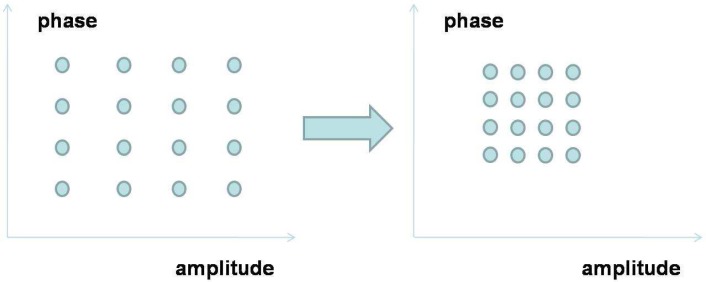
Illustration of parallel CUSUM test with successive refinement.

**Figure 41.**
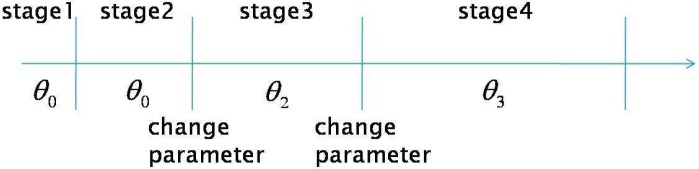
Illustration of multiple stages in successive refinement.

One disadvantage of the parallel CUSUM test is that the parameter candidates do not change throughout the test. When there are sufficiently many observations (e.g., when the threshold is large), the parameters can be almost perfectly estimated and then be applied to the quickest detection. Therefore, it is more reasonable to estimate the parameters simultaneously and change the set of parameter candidates. As illustrated in Figure 40, when we have more precise estimation for the parameters, we can narrow down the range of parameter candidates. Based on this philosophy, *successive refinement* is proposed in [152], in which the test is divided into multiple stages. In stage 1, parallel CUSUM test is applied with threshold *γ*_1_. When the corresponding stopping time is reached, we have some confidence on the distribution change. Then, we begin stage 2 during which parallel CUSUM test (with threshold *γ*_2_ > *γ*_1_) and parameter estimation are simultaneously carried out. When stage 2 is completed (the corresponding stopping time is reached), we use the parameter estimate to narrow down the range of parameter candidates and begin stage 3. Such a test-while-estimate procedure is repeated for certain times and then we claim that the change has happened. Such a successive refinement procedure is illustrated in Figure 41.

#### Performance Analysis

When the threshold is not large, performance metrics can be obtained via numerical simulations. For large threshold case (this is reasonable since we want to keep a low false alarm rate), an effective approach for analyzing the performance metrics is to apply Brownian motion approximation. Notice that

(63) $$B(t)=\frac{1}{\sqrt{N}}\sum_{n=1}^{Nt}L(n)$$

converges to a Brownian motion as *N* → ∞ when the distribution remains *p*_1_ or *p*_0_. Then, by applying the theory of Brownian motion, we can obtain the detection delay ARL, which is given by

(64) $$d\approx \frac{\gamma }{D\left(P_{1}||P_{0}\right)}$$

where *D*(*P*_1_||*P*_0_) is the Kullback-Leibler distance between *P*_1_ and *P*_0_. For the DTV case, the detection delay ARL is explicitly given by

(65) $$d\approx \frac{4\gamma \sigma_{n}^{2}}{A^{2}}$$

For the false alarm ARL *f*, we can obtain that *f* increases exponentially with respect to *γ*, i.e.,

(66) $$f=O\;(e^{\gamma })$$

#### Collaborative Quickest Spectrum Sensing

When multiple secondary users collaborate for spectrum sensing, they can exchange their observations to enhance the reliability and agility. One difficulty is to tackle the delay incurred by communications since the quickest detection is real-time. We consider a two-node (denoted by *A* and *B*) case and assume that the transmission of an observation needs *D* time slots. In [153], a two-thread CUSUM test is proposed to tackle the communication delay, in which the stopping time claiming the change is the earlier one of two stopping times:

(67) $$T_{A}^{*}=\text{min}\;\left(T_{A}^{\alpha },\;T_{A}^{\beta }\right)$$

where the stopping times $T_{A}^{\alpha }$ and $T_{A}^{\beta }$ are defined as follows (note that we use subscript *A* and *B* to distinguish the two nodes):

- Stopping Time $T_{A}^{\alpha }:T_{A}^{\alpha }$ can be obtained similarly to CUSUM test, namely
  (68) $$T_{A}^{\alpha }=\text{inf}\;\left\{t|m_{A}^{\alpha }\left(t-D\right)+\sum_{r=t-D+1}^{t}L_{A}(r)\geq \gamma_{A}\right\}$$
  where
  (69) $$m_{A}^{\alpha }(t)=\text{max}\;\left\{m_{A}^{\alpha }\left(t-1\right)+L_{A}(t)+L_{B}(t),\;0\right\}$$
- Stopping Time $T_{A}^{\beta }$: For $T_{A}^{\beta }$, only observations at node A during time slots *t* − *D* + 1 to *t* can be used. Then $T_{A}^{\beta }$ can be written as
  (70) $$T_{A}^{\beta }=\text{inf}\;\left\{t|\underset{t-D+1\leq k\leq t}{\text{max}}\sum_{r=k}^{t}L_{A}(r)\geq \gamma_{A}\right\}$$

The detailed explanation for the two stopping times and the extension to multiple nodes can be found in [153, 154].

### 9.2. Multiple Channels and Bayesian Case with Partial Observation

When there are multiple frequency channels (e.g., in DTV systems, there are multiple available frequency bands; if wider frequency band, e.g., 1000 MHz, is open for cognitive radio systems, we can divide the wide band into multiple frequency bins and consider each bin as a channel). It may be difficult for the secondary user to monitor all these channels since it requires high sampling rate and may not be supported by current commercial analog-digital converters (ADC). Therefore, the secondary user can monitor a subset of channels simultaneously and needs to jump across different channels. For simplicity of analysis, we assume that only one channel can be monitored at a time.

For simplicity, we consider *M* channels and assume that the secondary user can monitor one channel at a time. We denote the observation distributions over channel *m* with and without primary user by *P*_1*m*_ and *P*_0*m*_, respectively. Again, we assume that there are no primary users over the *M* channels at the beginning. At time slot *t*, primary user may emerge over channel *m* with *a priori* probability *ρ*(1 − *ρ*)^*t*−1^ (*ρ* is known to secondary users; thus the quickest detection is Bayesian) and the activities over different channels are mutually independent (note that the geometrical distribution of primary user emergence coincides with a two-state Markov model in which the probability of transmitting from idle to busy is *ρ*). We also assume that it requires *d_s_* time slots for switching between two channels.

#### Elements of Markov decision process

For such a Bayesian quickest spectrum sensing, we can apply the framework of Markov decision process, whose elements are given below

**Figure 42.**
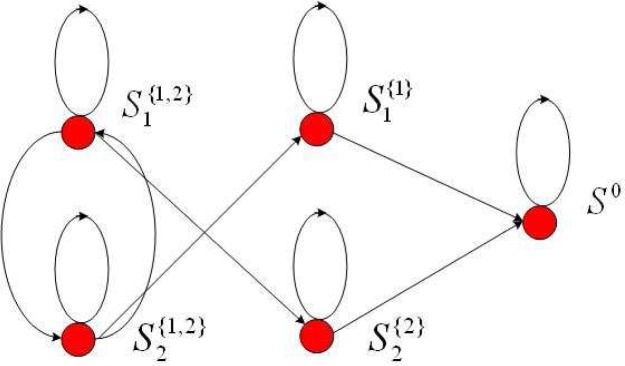
State transition diagram when *M* = 2.

**Figure 43.**
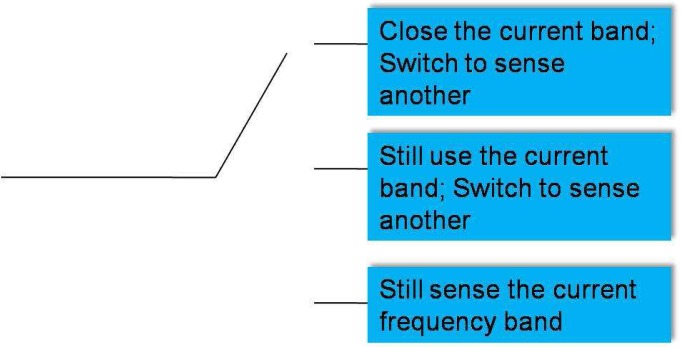
Three possible actions for each state.

- State space: we denote a state by $S_{m}^{\Omega }$, where Ω denotes the set of bands being used for data communication and *m* ∈ Ω denotes the band being sensed. When Ω is an empty set, the state, denoted by *S*^0^, means that all frequency bands are not being used by the secondary user. The state transition diagram for the case of *M* = 2 (2 channels) is illustrated in Figure 42. For example, the transition from state $S_{1}^{\left\{1,2\right\}}$ to state $S_{2}^{\left\{2\right\}}$ means that the secondary user claims that primary user has emerged over channel 1, stops communication over channel 1 and then monitor channel 2; the transition from state $S_{1}^{\left\{1,2\right\}}$ to state $S_{2}^{\left\{1,2\right\}}$ means switching to monitor channel 2 without stopping the communications over channel 1.
- Action space: for each state (except *S*^0^), the secondary user can take three types of actions, which are illustrated in Figure 43, namely continuing transmitting and monitoring the current channel, switching to monitor another channel without stopping the communication over the current channel, and switching to monitor another channel while stopping the communication over the current channel.
- Cost function: we define a single cost function which leads to the corresponding optimal control policy. As mentioned before, we need to consider the penalties incurred by both detection delay and false alarm. Therefore, we define the cost function as follows (note that all the probabilities are conditioned on the observations; for simplicity, we ignore the condition in the expression of probabilities):
  $$\begin{array}{lll} R & = & \sum_{m=1}^{M}P\left(T_{m}>T_{m}^{*}\right)+c\sum_{m=1}^{M}E\left[{\left(T_{m}^{*}-T_{m}\right)}^{+}\right]\\ & = & \sum_{m=1}^{M}P\left(T_{m}>T_{m}^{*}\right)+c\sum_{m=1}^{M}E\left[\sum_{k=1}^{T_{m}^{*}-1}P\left(T_{m}\leq k\right)\right] \end{array}$$
  where *c* is a weighting factor balancing the penalties from detection delay and false alarm and subscript *m* denotes the index of channels (recall that T* means the time claiming the emergence of primary user and *T* is the actual emergence time). Therefore, for time slot *k* (suppose that the current channel is *m*), we have
  – if not stopping communication over the current channel, we get penalty *cP*(*T_m_* < *k*) (detecting delay);
  – if stopping transmission over the current channel, we get penalty $P\left(T_{m}>T_{m}^{*}\right)$ (false alarm).
- Control policy: we consider a stationary control policy, i.e. the action is dependent on only the current state and is independent of time.

#### Dynamic Programming

It is well known that the optimal control policy of Markov decision process can be solved by dynamic programming [155, 156, 157]. A general formulation for a Markov decision process is

(71) $$s_{t+1}=f\left(s_{t},\;u_{t},\;w_{t}\right)$$

where *s_t_* is the state at time *t*, *u_t_* is the control policy and *w_t_* is random perturbation. Suppose that the corresponding cost function is given by

(72) $$J=\sum_{t=1}^{\Gamma }E\left[g\left(s_{t},\;u_{t},\;w_{t}\right)\right]$$

where *g* is the function of cost for each time slot and Γ is the final time slot (here we consider finite horizon case).

A fundamental concept in dynamic programming is cost-to-go function (also called value function if we use reward instead of cost), which is defined as the sum of cost from time *t* to Γ, denoted by *J_t_*(*s*) when the current state is *s*, i.e.

(73) $$J_{t}\;(s)=\sum_{\tau =t}^{\Gamma }E\left[g\left(s_{\tau },\;u_{\tau },\;w_{\tau }\right)|s_{t}=s\right]$$

With the tool of cost-to-go functions, we can obtain that the optimal control policy must satisfy the Bellman’s equation, which is given by

(74) $$J_{t}^{*}(s_{t})=\underset{u_{t}}{\text{min}}\;E\left[c\left(s_{t},\;u_{t},\;w_{t}\right)+J_{t+1}^{*}\left(s_{t+1}\right)\right]$$

The corresponding optimal control policy is obtained via

(75) $$\mu_{t}^{*}=\text{arg}\;\underset{u_{t}}{\text{min}}\;E\left[c\left(s_{t},\;u_{t},\;w_{t}\right)+J_{t+1}^{*}\left(s_{t+1}\right)\right]$$

The computation of cost-to-go functions is in a backward order. At the beginning, we compute the cost-to-go function at the final time slot Γ. The computation is straightforward since it is a one-snapshot optimization and need not look into the future. Then, we substitute $J_{\Gamma }^{*}(s)$ into the right hand side of (74) and carry out optimization for $J_{\Gamma -1}^{*}\;(s)$. Once we obtain $J_{\Gamma -1}^{*}\;(s)$, we can compute $J_{\Gamma -2}^{*}\;(s)$. We repeat the procedure until we obtain $J_{1}^{*}\;(s)$ and consequently the whole optimal control policy.

#### Finite Horizon Case

Now, we can apply the powerful tool of dynamic programming to our problem. We first consider finite horizon case, i.e., we consider a time window [1,Γ] and close the communications over all channels after time Γ. Then, it is easy to verify that

(76) $$J_{\Gamma }\;\left(S_{m}^{\Omega }|X_{0}^{\Gamma }\right)=\sum_{m\in \Omega }P\left(T_{n}>\Gamma \right)$$

i.e., the sum of false alarm probabilities for the remaining active channels. For 1 ≤ *t* < Γ, we can apply the Bellman’s equation to compute the cost-to-go function $J_{t}\left(S_{m}^{\Omega }|X_{0}^{t}\right)$. The details can be found in [158].

One problem with the above approach of dynamic programming is that we need to record all observations $X_{0}^{\Gamma }$, which requires prohibitively large memory. Fortunately, we can show that the *a posteriori* probabilities ${\left\{P\left(T_{m}\leq t|X_{0}^{t}\right)\right\}}_{m=1,\ldots M}$ are sufficient statistics for the cost-to-go functions, i.e., for time slot *t*, the cost-to-go function $J_{t}\;\left(S_{m}^{\Omega }|X_{0}^{t}\right)$ can be written as $J_{t}\;\left(S_{m}^{\Omega }|p_{t}\right)$, where

(77) $${\left(p_{t}\right)}_{m}=P\left(T_{m}\leq t|X_{0}^{t}\right)$$

Therefore, we need to record and update only the *M*-vector p*_t_*, which requires only constant amount of memory. The *a posteriori* probabilities can be computed in a recursive manner (the recursive expression can be found in [158]), thus being quite efficient. Note that each cost-to-go function is a function of p*_t_*, instead of a constant. Therefore, in numerical computation of the cost-to-go functions, we need to discretize the *M*-vector p*_t_* first.

#### Infinite Horizon Case

A drawback of the finite horizon case is that we need to compute a cost-to-go function for each combination of time and state. For the case of *M* = 2, we have four non-trivial states. Therefore, if we consider a time window of 500 time slots, we need to compute 2000 cost-to-go functions, which brings substantial computational cost to the system. Meanwhile, the assumption of finite horizon is unreasonable since the spectrum sensing may last for any arbitrarily long period of time.

Therefore, it is more desirable to study the infinite horizon case, i.e., Γ → ∞. An advantage of considering infinite horizon case is that we can ignore the subscript of time in the cost-to-go functions because it is easy to show that, as Γ → ∞,

(78) $$J_{t}\left(S_{m}^{\Omega }|p_{t}\right)\to J\left(S_{m}^{\Omega }|p_{t}\right),\;\;\;\;\;\;\;\;\;\;\;\;\;\;\;\forall t$$

By considering infinite horizon, the number of cost-to-go functions is reduced to 4 when *M* = 2. We can further simplify the cost-to-go functions using the following two features (the details can be found in [158]):

- **Symmetry** : frequency bands are symmetric, the permutation of the frequency bands yields the same cost-to-go function.
- **Argmin**: If transiting to another frequency band, the secondary node should always choose the frequency band having the largest *a posteriori* probability.

After simplifying the cost-to-go functions, we can apply the Bellman’s equation to compute the optimal cost-to-go functions and the corresponding optimal control policy.

#### Combating the Curse of Dimensions

Although we have simplified the cost-to-go functions, the number of states still becomes intolerably large when *M* becomes large. The discretization of the *M*-vector p*_t_* adds more dimensions to the cost-to-go functions. Such a curse of dimensions is an inherent difficulty for Markovian decision process. Therefore, it is desirable to apply techniques in approximate dynamic programming such as Rollout, approximate cost-to-go function, open look feedback control or model prediction control.

In [156], two simple principles are applied to obtain simplified control policy:

- Limited lookahead policy (LLP): in standard dynamic programming, the optimal control policy needs to look into the future; we can relax this requirement and look ahead for only limited time slots.
- Certainty equivalent control (CEC): we can replace the random variables in the optimization problem with their expectations. Surprisingly, such an operation still yields the optimal control policy for linear control problems. Since our problem is non-linear, the resulted control policy is suboptimal.

By using the LLP principle, we consider only two most ‘dangerous’ channels, i.e., the channel being monitored and the channel having the largest *a posteriori* probability that the primary user has emerged among the channels not being sensed (for simplicity, we assume that they are channel 1 and channel 2). Then, we reduce the problem to a much simpler case of *M* = 2. By applying the CEC principle, we compute the expected time of primary user emergence over channels 1 and 2, denoted by *T̄*_1_ and *T̄*_2_. Since we need to consider the impact of false alarm and transition time *d_s_*, we compensate these two expectation by

(79) $${\tilde{T}}_{1}^{t}={\tilde{T}}_{1}^{t}+\frac{1}{c}\left(1-{\left(p_{t}\right)}_{1}\right)$$

and

(80) $${\tilde{T}}_{2}^{t}={\tilde{T}}_{2}^{t}+\frac{1}{c}\left(1-{\left(p_{t}\right)}_{2}\right)+d_{s}$$

Then, we consider the compensated times as their true values. A heuristic decision rule is given by

**Figure 44.**
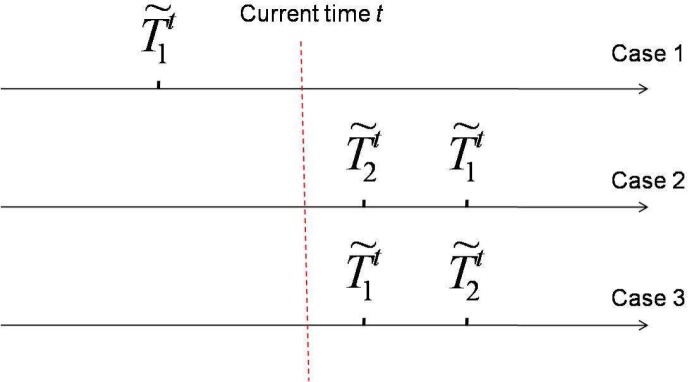
Illustration of three cases in the heuristic decision rule.

- If ${\tilde{T}}_{1}^{t}\leq t$, stop the communication over channel 1 (the current channel) and switch to channel 2;
- If ${\tilde{T}}_{1}^{t}\geq {\tilde{T}}_{2}^{t}\geq t$, stop sensing channel 1 and switch to sense channel 2.
- If ${\tilde{T}}_{2}^{t}\geq {\tilde{T}}_{1}^{t}\geq t$, continue to sense channel 1;

The three cases are illustrated in Figure 44.

## 10. Soft Decision Cognitive Radio and Hybrid Overlay/Underlay Cognitive Radio Waveform Design

### 10.1. Overview

Here, we present a novel soft decision cognitive radio paradigm to combine the benefits of underlay CR and overlay CR to maximize the channel capacity and spectrum efficiency. Specifically, the soft decision CR will detect not only if one spectrum block is used or unused as in current spectral sensing, but detect if it is *underused*. Moreover, the instantaneous interference tolerance level of all underused bands will be determined by weighted spectrum estimate (WSE). Based on the instantaneous interference tolerance level, we employ a soft decision spectrally modulated spectrally encoded (SMSE) framework to design hybrid overlay/underlay waveform to distribute transmission power over the entire bandwidth including both unused bands and underused bands to maximize the channel capacity. Current overlay CR and underlay CR can be viewed as two extreme cases of the general soft decision cognitive radio paradigm.

### 10.2. Underlay CR and Overlay CR

**Figure 45.**
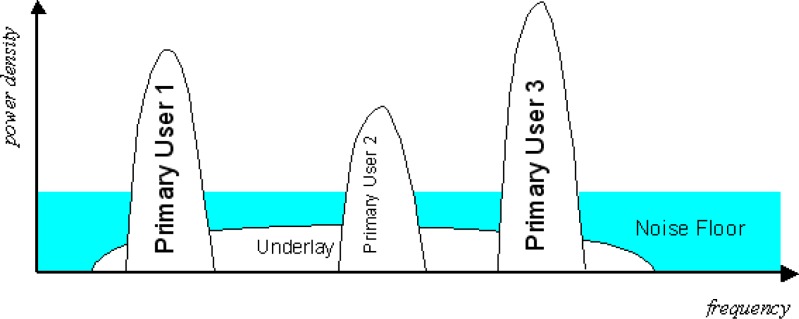
Underlay CR transmission.

**Figure 46.**
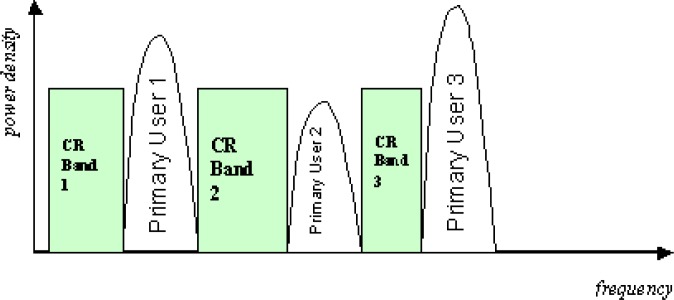
Overlay CR transmission.

Figures 45 and 46 illustrate the concepts of underlay CR transmission and overlay CR transmission. We start with the famous Shannon channel capacity equation:

(81) $$C=W\;\text{log}\;\left(1+\frac{S}{N}\right)$$

It is well known that to increase the channel capacity in a communication system, we need to increase the SNR (signal to noise ratio) $\frac{S}{N}$ or the bandwith *W*, or both.

In underlay CR transmission, a very large contiguous bandwidth is used for secondary user’s transmission, with the primary users operating within the same bandwidth. In this way, we maximize the bandwidth *W* in (81). However, to avoid interferences to primary (licensed) users, the underlay CR transmission has to limit its transmission power density at a very low level. Hence, the channel capacity of underlay CR transmission is extremely limited although it has maximized the transmission bandwidth. Specifically, the channel capacity of underlay CR transmission is

(82) $$C_{\mathrm{Underlay}}=W\;\text{log}\;\left(1+\frac{\Phi_{\mathrm{Underlay}}\;W}{n_{0}W+\sum_{i=1}^{M}\Phi_{p_{i}}\;W_{p_{i}}}\right)$$

where *n*_0_ is the additive Gaussian noise power spectrum density, Φ*_Underlay_* is the average power spectrum density of underlay CR transmission, *M* is the total number of primary users operating within the bandwidth *W*, Φ*_pi_* is the average power spectrum density of the *i^th^* narrowband primary user’s transmission and *W_pi_* is the corresponding bandwidth of *i^th^* primary user. Notice that underlay CR transmission suffers interference from all primary users, which is characterized by $\sum_{i=1}^{M}\Phi_{p_{i}}\;W_{p_{i}}$. Since (1) the power spectrum density of underlay CR transmission Φ*_Underlay_* is very low and (2) the primary narrowband users have much higher power spectrum density Φ*_pi_*, the signal to interference and noise ratio (SINR) is significantly decreased. As a direct result, even though underlay CR transmission has a very large bandwidth, its channel capacity is very limited.

On the other hand, overlay CR transmission finds the unused frequency bands and only transmits over those bands, totally avoiding interference to primary users. The channel capacity of cognitive radio transmission is characterized as

(83) $$C_{\mathrm{Overlay}}=\sum_{k=1}^{N}W_{u_{k}}\;\text{log}\;\left(1+\frac{\sum_{k=1}^{N}\Phi_{CR_{k}}\;W_{u_{k}}}{n_{0}\sum_{k=1}^{N}W_{u_{k}}}\right)$$

where *N* is the total number of unused bands in the entire bandwidth *W*, *W*_*u*_*k*__ is the bandwidth of the *k^th^* unused band, Φ_*CR*_*k*__ is the power spectrum density of cognitive radio transmission on the *k^th^* unused band. It is evident that in cognitive radio transmission, the total bandwidth exploited is less than the total bandwidth *W*. However, since (1) there is no interference from primary users to cognitive radio and (2) there is no limit in the cognitive radio transmission power spectrum density Φ_*CR*_*k*__, the signal to noise ratio is much improved (compared to underlay CR transmission). As a direct result, the channel capacity of cognitive radio is much higher than that of underlay CR transmission.

### 10.3. Soft Decision Cognitive Radio

**Figure 47.**
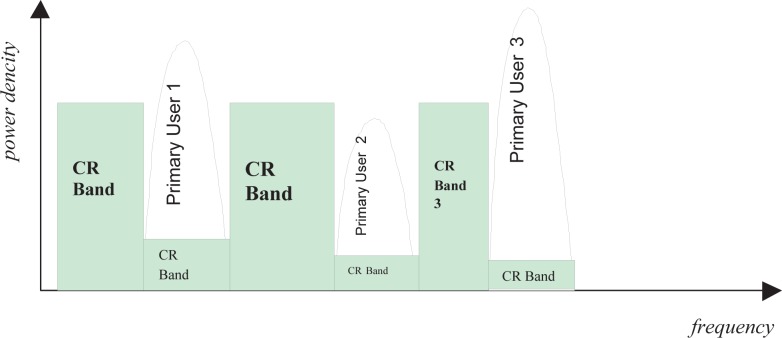
Soft decision cognitive radio.

In cognitive radio, the transmitter continuously monitors the radio spectrum and dynamically identify frequency bands into two categories: used bands or unused bands. In other words, the cognitive radio makes the usability of one frequency band by employing a hard decision based on spectrum sensing result. However, the coexistence of underlay CR transmission and primary users indicates that all the primary users’ transmissions can tolerate some level of interference. Hence, we can further increase the channel capacity of cognitive radio by making a soft decision on the usability of each and every used band. If we can determine the interference tolerance level of each primary user, the cognitive radio can transmit over both the unused bands and the used bands to optimize the spectrum usage and maximize the channel capacity. We name this system Soft Decision Cognitive Radio. Figure 47 shows such a system.

Assume the interference tolerance level (the maximum allowed interference power spectrum density) of the *i^th^* used band is *I_i_*. Current cognitive radio assumes that *I_i_* = 0, i.e., no transmission is allowed if the band is being used. Employing knowledge of the interference tolerance level *I_i_*, the channel capacity of such a proposed system is

(84) $$C_{\mathrm{new}}=W\;\text{log}\;\left(1+\frac{\sum_{k=1}^{N}\Phi_{C{R1}_{k}}\;W_{u_{k}}+\sum_{i=1}^{M}\Phi_{C{R2}_{i}}\;W_{p_{i}}}{n_{0}\;W+\sum_{i=1}^{M}\Phi_{p_{i}}\;W_{p_{i}}}\right)$$

where Φ_*CR*1_*k*__ is the cognitive radio transmission power spectrum density on *k^th^* unused band, and Φ_*CR*2_*i*__ is the cognitive radio transmission power spectrum density on *i^th^* used band.

To maximize the channel capacity of the proposed system, we need to maximize *C_new_* subject to the following constraints:

(85) $$\begin{array}{ll} \Phi_{C{R2}_{i}} & \leq I_{i},\;\forall i\\ \Phi_{C{R1}_{k}} & \leq \phi_{k},\;\forall k \end{array}$$

(86) $$\sum_{k=1}^{N}\Phi_{C{R1}_{k}}\;\;\;W_{u_{k}}+\sum_{i=1}^{M}\Phi_{C{R2}_{i}}\;W_{p_{i}}\leq S$$

where *I_i_* is the interference tolerance level at *i^th^* used band, *ϕ_k_* is the maximum allowed transmission power spectrum density regulated by FCC at *k^th^* unused band, *S* is the total transmission power.

Since the number of unused bands *N* and the number of used bands *M* are not gigantic numbers, this optimization is relatively small scale and a Lagrange multiplier method with numerical optimization could quickly generate a solution.

It is evident that current overlay CR transmission and underlay CR transmission are just two special cases of the general soft decision cognitive radio paradigm: if we force *I_i_* = 0, the system reduces to current cognitive radio; if we force *I_i_* and *ϕ_k_* to be FCC UWB spectrum mask, the system reduces to underlay CR transmission.

**Figure 48.**
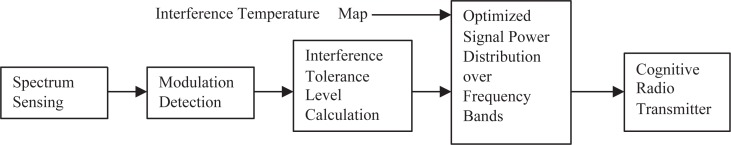
Cognitive radio.

Figure 48 illustrates a block diagram of the proposed soft decision cognitive radio system:

### 10.4. Hybrid Overlay/Underlay Waveform Design for Soft Decision Cognitive Radio

Previous work provides a general analytic framework for SMSE signals that accommodates multi-carrier, CR-based waveforms [159]. Specifically, an arbitrary CR waveform can be expressed in terms of its amplitude (**A**), phase (**Θ**) and frequency (**F**) characteristics. These three factors aid in SMSE waveform design through six design variables, namely data modulation (**d**), Code (**c**), window (**w**), orthogonality (**o**) and two frequency allocation variables. An in-depth treatment of the SMSE analytic development and the family of SMSE waveforms is provided in [159, 160]. Considering *N_f_* total frequency components, the coding **c** = [*c*_1_,*c*_2_,...,*c*_*N*_*f*__], *c_i_* ∈ 𝔺, data modulation, **d** = [*d*_1_,*d*_2_,...,*d*_*N*_*f*__], *d_i_* ∈ 𝔺, and windowing, **w** = [*w*_1_,*w*_2_,...,*w*_*N*_*f*__], *w_i_* ∈ 𝔺 vectors account for component-by-component amplitude and/or phase variations. A phase only variable ∅ = [*o*_1_,*o*_2_,...,*o*_*N*_*f*__],*o_i_* ∈ 𝔺 is used for orthogonality between symbol streams and facilitate multiple access.

The analytic SMSE framework development begins by considering data, code and window variables. The *m^th^* frequency component of the *k^th^* symbol is given by

(87) $$S_{k}\;\left[m\right]=c_{m}\;d_{m,k}\;w_{m}\;e^{j\left(\theta_{d_{m,k}}+\theta_{c_{m}}+\theta_{w_{m}}\right)}$$

where *m* = 0,1,...,*N_F_* − 1 is the frequency index and *c_m_*, *d_m_* are magnitude and phase design variables.

The expression in (87) is next modified to incorporate frequency and orthogonality variables. Frequency component selection is a function of two factors, including an *available* variable a = [*a*_1_,*a*_2_,...,*a*_*N*_*f*__], *a_i_* ∈ {0, 1} and a *use* variable u = [*u*_1_,*u*_2_,...,*u*_*N*_*f*__], *u_i_* ∈ {0, 1}. Given an *N_f_*-point fast Fourier transform (FFT) process, *N_f_* frequency components or spectral bands are available for waveform design. It is important to note that the frequency assignment variable takes on binary values 0 or 1 to indicate the spectrum availability for secondary users. As a direct result, this pool of frequencies is reduced by component selection to create a number of CR available frequencies *and* usable frequencies. The *m^th^* component of the *k^th^* CR symbol corresponds to

(88) $$S_{k}\left[m\right]=a_{m}\;u_{m}\;c_{m}\;d_{m,k}\;w_{m}\;e^{j\left(\theta_{d_{m,k}}+\theta_{c_{m}}+\theta_{w_{m}}+\theta_{o_{m,k}}\right)}$$

where the product *a_i_ u_i_* ∈ {0, 1}. The discrete time domain SMSE waveform is obtained by taking the Inverse Discrete Fourier Transform (IDFT) of (88) according to

(89) sk[n]=1Nf Re {∑m=0Nf−1am um cm dm,k wm ej(2πfm tn+θdm,k+θcm+θwm+θom,k)}

where *t_k_* ≤ *t_n_* ≤ *t_k_* + *T*, *f_m_*= *f_c_* + *m*Δ*f*, *T* is the symbol duration and Δ*f* = 1/*T* is the frequency resolution [159].

The SMSE framework provides a unified expression for generating and implementing a host of multi-carrier type waveforms (e.g., OFDM [161], MC-CDMA [162], CI/MC-CDMA [163, 164], TDCS [165, 166], etc.) and satisfies current CR goals of exploiting unused spectral bands. However, it does not exploit *underused* spectrum. This section re-visits the original SMSE framework development and the frequency assignment variables to exploit both *unused* and *underused* spectrum to generate both overlay-CR and underlay-CR type waveforms.

**Figure 49.**
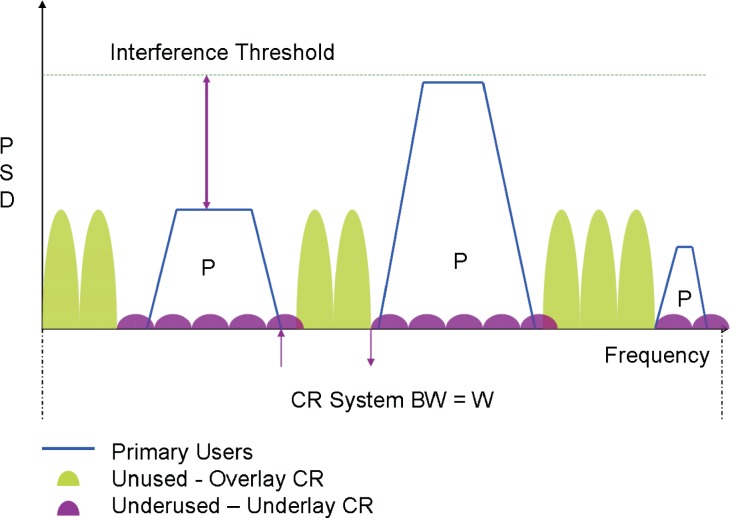
Identification of primary users, unused and underused spectral region .

Figure 49 illustrates a conceptual view of the unused and *underused* spectrum utilization using an arbitrary interference threshold (IT). IT is assumed to be a limit set forth by the primary users based on the measured power spectrum density in a given bandwidth. Two cases of under utilized spectrum are demonstrated: (1) when the spectral assignment is based on a binary decision, the bands adjacent to the primary users are unavailable to overlay-CR users and (2) primary users bands below the IT are unavailable to CR users. A soft decision CR (SDCR) will be able to exploit these *underused* frequency bands to improve spectral efficiency and increase channel capacity. To support the envisioned SDCR system, the original SMSE framework is extended to account for both *unused* and *underused* frequency bands.

**Figure 50.**
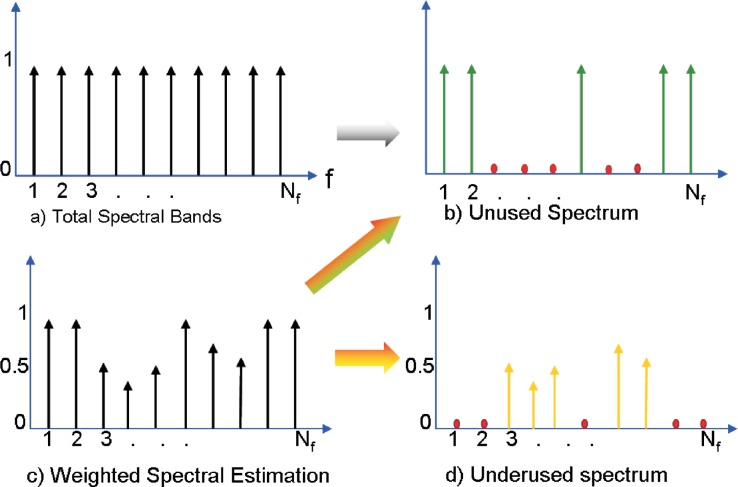
Spectrum parsing using weighted spectrum estimation in realization of SD-SMSE waveform.

**Figure 51.**
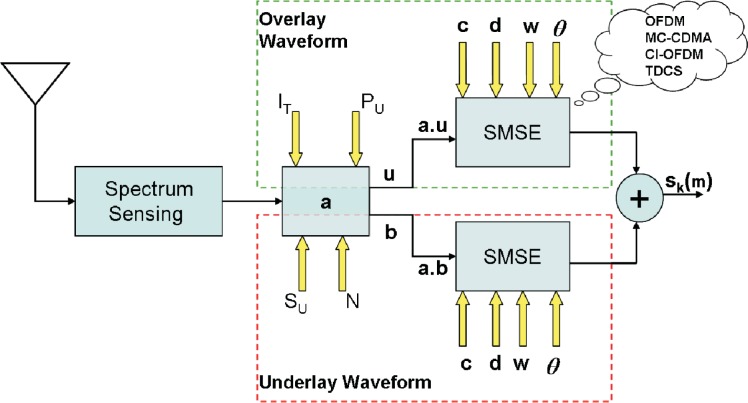
Block diagram representation of SD-SMSEframework [167].

The proposed SD-SMSE framework is first illustrated using Figures 50 and 51, then the design variables are re-defined to extend the SMSE expression to account for both unused and *underused* spectrum. Figure 50a,b shows how the current CR framework identifies the used and unused spectrum based on binary decisions. Figure 50c shows the weighted spectrum estimation resulted from spectrum sensing block in Figure 51. The weighted spectrum estimate (WSE) (**a**) is further processed by taking into account inputs from the IT estimator, primary users, other secondary users requirements and channel conditions. Specifically, the weighted spectrum estimate provides a metric of the allowable transmission power density at each and every frequency component in the entire bandwidth. Hence, the WSE divides the entire bandwidth into unused (**u**) and *underused* (**b**) frequency components and both the unused and *underused* spectrum can be exploited. Notice in Figure 50 that different *underused* frequency components have different allowable CR transmission power densities. It is envisioned that a CR-based SDR will have the option to choose an overlay-CR, underlay-CR or hybrid overlay/underlay waveform to improve performance based on the scenario, situation and need.

The first step in SD-SMSE framework development is to re-examine the design variables in the original SMSE framework. For the SD-SMSE development, frequency related factors are termed primary variables, whereas amplitude and phase related factors are termed secondary variables. Since the objective here is to optimize the spectrum usage, only frequency components related design variables are considered. From this point forward the SD-SMSE framework development is based on the scenario depicted in Figure 50. As shown in Figure 50c, the weighted spectrum estimate represents all frequency components, which can be utilized for secondary user applications. It is represented by variable a with the range changed from binary values (hard decision) to real values (soft decision), i.e.,

(90) $$a=\left[a_{0},a_{1},\ldots ,a_{N_{f}-1}\right],\;0\leq a_{m}\leq 1$$

From the weighted spectrum estimate a, the *unused* spectrum vector u can be derived as

(91) $$u=\left[u_{0},\;u_{1},\ldots ,u_{N_{f}-1}\right]$$

where,

(92) $$u_{m}=\{\begin{array}{ll} 1 & i\;f\;a_{m}=1\\ 0 & \mathrm{else} \end{array}m=0,1,\ldots N_{f}-1$$

The original SMSE hard decision CR design transmits over the unused spectrum specified by u. Now introducing a new design variable b to account for the *underused* spectrum,

(93) $$b=\left[b_{0},b_{1},\ldots b_{N_{f}-1}\right]$$

where

(94) $$b_{m}=\{\begin{array}{ll} 0 & a_{m}=1\\ a_{m} & a_{m}\neq 1 \end{array}$$

for *m* = 0,1,…,*N_f_* − 1. Note that when *a_m_* = 1 the value of *b_m_* = 0. This is because when *a_m_* = 1, the spectral component is *unused* and accounted for in the assignment of *u_m_*. It is obvious that if one frequency component is *underused*, it cannot also be counted as unused and vice versa, i.e., *u_m_* = 0 if *b_m_* > 0 and *b_m_* = 0 if *u_m_* = 1.

The remaining waveform design variables, i.e., code (c), data (d), window (w) and orthogonality (o), remain unchanged from the original SMSE framework.

Applying all these design variables, the *m^th^* component of the *k^th^* data symbol of the SD-SMSE can be expressed as

(95) $$S_{k}\left[m\right]=a_{m}\;c_{m}\;d_{m,k}\;w_{m}\;e^{j\left(\theta_{d_{m,k}}+\theta_{c_{m}}+\theta_{w_{m}}\theta_{o_{m,k}}\right)}=\{\begin{array}{ll} u_{m}\;c_{m}\;d_{m,k}\;w_{m}\;e^{j\left(\theta_{d_{m,k}}+\theta_{c_{m}}+\theta_{w_{m}}\theta_{o_{m,k}}\right)} & a_{m}=1\\ b_{m}\;c_{m}\;d_{m,k}\;w_{m}\;e^{j\left(\theta_{d_{m,k}}+\theta_{c_{m}}+\theta_{w_{m}}\theta_{o_{m,k}}\right)} & a_{m}\neq 1 \end{array}$$

The expression in (95) can be decomposed into *unused* and *underused* SMSE waveform representing the new SDCR architecture shown in Figure 51. Applying the IDFT to (95) results in the discrete time domain waveform given by

(96) sk[n]=1Nf Re {∑m=0Nf−1am cm dm,k wm ej(2πfmtn+θdm,k+θcm+θwm+θom,k)}

(97) sk[n]=1Nf Re {∑m=0Nf−1um cm dm,k wm ej(2πfmtn+θdm,k+θcm+θwm+θom,k)}+1Nf Re {∑m=0Nf−1bm cm dm,k wm ej(2πfm tn+θdm,k+θcm+θwm+θom,k)}

where the first summation in (97) represents the *unused* frequency components and the second summation accounts for *underused* frequency components.

The SMSE expression in (96) was demonstrated by applying it to a number of OFDM based multi-carrier signals [159, 168, 169]. The process of generating these waveforms can be viewed as a two step approach: (1) generating the frequency related primary variables, and (2) applying the secondary variables such as the code code, data modulation, windowing and orthogonality to the frequency vector. Since the SD-SMSE only focused on manipulating the primary variables, all of the OFDM based multi-carrier modulations expression such as NC-OFDM, NC-MC-CDMA, NC-CI/MC-CDMA and NC-TDCS are applicable to both overlay-CR and underlay-CR scenarios.

### 10.5. SD-SMSE Overlay Waveform

Current overlay CR transmission employs a waveform to exploit unused spectral bands and thus represent a special case (subset) of SDCR with no *underused* frequency components being exploited. In the SMSE framework, forcing the *underused* variable b to be zero and the frequency assignment variable a to take on binary values results in,

(98) b=[0,0,…,0]

(99) $$a=\left[a_{0},a_{1},\ldots ,a_{N_{f}-1}\right],\;a_{m}\in \left\{0,1\right\}$$

where the second summation in (97) is eliminated and reduces to current hard decision CR overlay:

(100) sk[n]=1Nf Re {∑m=0Nf−1um cm dm,k wm ej(2πfm tn+θdm,k+θcm+θwm+θom,k)}

### 10.6. SD-SMSE Underlay Waveform

Unlike overlay-CR waveforms that only operate in unused spectrum bands, underlay-CR waveform operates in *underused* spectrum regions. An underlay-CR waveform spreads its signal over a wide band-width to minimize interference to existing primary users and to achieve the required processing gain to improve its own performance. Underlay-CR approaches have been generally associated with UWB technology. By definition, a signal is defined as UWB if it occupies a bandwidth that is greater than 500 MHz. Therefore, not all underlay-CR waveforms can be classified as UWB per this definition. For example, a low data rate underlay waveform used as a control channel might only require a few mega hertz of bandwidth. In the SD-SMSE context, UWB is a special implementation of an underlay-CR waveform. An UWB transmission uses underlay waveform which operates across all spectral components while minimizing interference to primary users by limiting its transmission power spectral density. Hence, its allowable transmission power spectral density is dictated by the primary user (among all those present) that is most sensitive to interference. In this case, all frequency components are treated as *underused* components. Hence, by setting

(101) u=[0,0,…,0]

(102) b=[K,K,…,K], 0<K<1

The first summation in (97) can be eliminated which results in a CR underlay waveform corresponding to an UWB transmission:

(103) sk[n]=1Nf Re {∑m=0Nf−1Kdm,k wm ej(2πfm tn+θdm,k+θcm+θwm+θom,k)}

where *K* is a constant obtained by taking the minimum value of the weighted power spectral density shown in Figure 50. Note that **b** was assumed to constant for simplicity purpose, in general each *underused* spectral components can have different spectral weights capable of employing adaptive baseband modulations.

### 10.7. Hybrid Overlay/Underlay

For the soft decision CR, the waveform achieves benefits of both overlay-CR and underlay-CR wave-forms by exploiting both *unused* and *underused* spectral regions. This is done by employing soft decision criteria at each distinct frequency component while minimizing the interference to primary users [170, 171, 172]. The expression in (97) represents the hybrid overlay/underlay waveform utilizing the SD-SMSE framework.

## 11. Vision and Future Work

There is a trend to integrate cognitive radio with cognitive radar, together with anti-jamming capabilities. The advent of multi-GHz arbitrary waveform generators and the need for cognitive radio make this integration attractive. The multi-GHz waveform provides super anti-jamming capabilities. The objective of this proposal is to investigate a novel paradigm of integrating the three ingredients; the multi-GHz waveform (through the use of revolutionary compressive sampling) is jointly considered with the dynamic spectrum access (through a novel system architecture for spectrum sensing). The primary challenge is caused by the wideband (multi-GHz) nature of the problem at hand.

One of the central tenets of communications is the Shannon/Nyquist sampling theory, which states that the number of samples required to capture a signal is dictated by its bandwidth. It is well known today, however, the Nyquist rate is a sufficient but by no means necessary condition. Compressive sampling or compressed sensing (CS) enables of the faithful recovery of signals, images, and other data, from what appear to be highly sub-Nyquist-rate samples. At the heart of the new approach are two crucial observations. (1) The Shannon/Nyquist signal representation exploits only minimal prior knowledge about the signal being sampled, namely its bandwidth. Most objects of our interest, however, are structured and depend upon a smaller number of *degrees of freedom* than the bandwidth suggests. In other words, most objects are *sparse* or *compressible* in the sense that they can be encoded with just a few numbers without much numerical or perceptual loss. (2) The useful information content in compressible signals can be captured via sampling protocols that directly condense signals into a small amount of data. In short, and in stark contrast with conventional wisdom, the theory of CS asserts that one can combine “low-rate sampling” with computation power for efficient and accurate signal acquisition.

On the other hand, at the heart of this cognitive radio, there is spectrum sensing: narrowband and wideband. The narrowband spectrum sensing—represented by IEEE 802.22—is mature and adopted by the Federal Communication Commission (FCC). The wideband spectrum sensing, in particular multi-GHz, seems be in its infancy. The FCC has abandoned the concept of interference temperature that may be a candidate. As a result, the proposed research may have potential impact on the future policy on spectrum sharing for wideband cognitive radio.

Roughly speaking, if there is *a prior* information of the primary radio such as modulation format, pilot, symbol rate, etc., spectrum sensing—that enables the secondary radio for dynamic spectrum access (DSA)—can be implemented using approaches such as matched filter, energy detection, cylcostationary sensing, eigenvalue based sensing, etc. For wideband (multi-GHz) spectrum sensing, however, there is no practical way to locate unused white spectra. Another critical challenge is wideband RF front-end capable of simultaneous sensing of several GHz wide spectrum.

It is critical to test key system components in different system settings. Three system models are proposed: (1) MATLAB/C simulation model, (2) waveform model, and (3) real-time FPGA system model. The majority of the research results are obtained in the domain of MATLAB/C simulation model. This approach is simple. But many real-world limitations cannot be simulated. The unique approach of this proposal is to combine these three models. Real-time FPGA model is the ultimate test, but time-consuming. We will, thus, use this model when the system concept is very stable. As a result, most system emulations are based on the waveform model. This waveform model is made available only recently, with the latest A/D conversion for 9.6 GHz signals with 10-bit resolution. The TTU’s lab is fortunate to be awarded the NSF MRI grant that makes this possible.

## 12. Conclusion

Dynamic spectrum access is a must-have ingredient for future sensors that are ideally cognitive. The goal of this paper is a tutorial treatment of wideband cognitive radio and radar—a convergence of (1) algorithms survey, (2) hardware platforms survey, (3) challenges for multi-function (Radar/Comms) multi-GHz front end, (4) compressed sensing for multi-GHz waveforms—revolutionary A/D, (5) machine learning for cognitive radio/radar, (6) quickest detection, and (7) overlay/underlay cognitive radio waveforms.

One focus of this paper is to address the multi-GHz wideband front end that is the challenge for the next-generation cognitive sensors. This unifying theme of this paper is to spell out the convergence for cognitive radio, radar, and electronic warfare.

The future work lies in two aspects: (1) multi-GHz wideband platforms, and (2) intelligently leaning algorithms. The first aspect requires new front end design. Compressive sampling is important in this context. The second aspect requires the integration of machine learning and artificial intelligence into communications and network. It is believed that networking for cognitive radio nodes is open: network testbed is required to gain more experimental knowledge—necessary for future rigorous science.

## Figures and Tables

**Figure 24.**
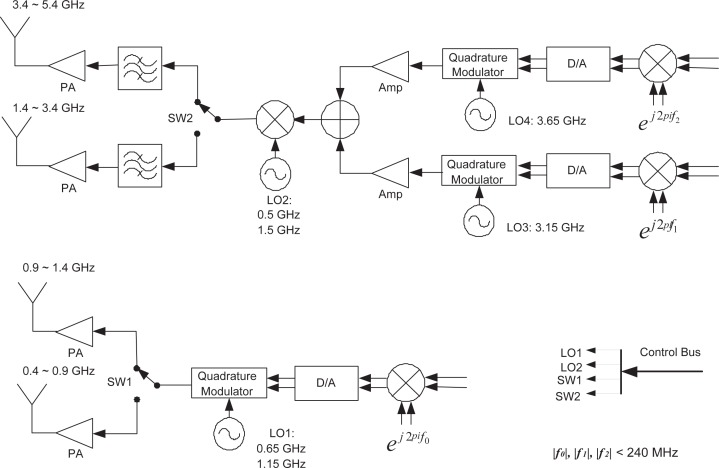
Transmitter front-end architecture.

**Figure 25.**
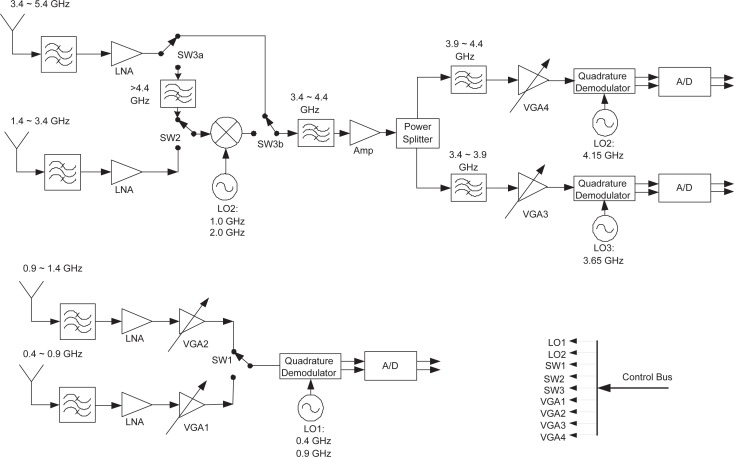
Receiver front-end architecture.

**Table 1.** USRP daughterboard list with specifications.

Board	Rx / Tx	Frequency Range	Tx Power
Basic Tx/Rx	Tx/Rx	1 MHz to 250 MHz	N/A
LF Tx/Rx	Tx/Rx	DC to 30 MHz	N/A
TVRX	Rx	50 MHz to 860 MHz	N/A
DBSRX	Rx	800 MHz to 2.4 GHz	N/A
WBX0510	Transceiver	50 MHz to 1 GHz	100mW (20dBm)
RFX900	Transceiver	750 to 1050 MHz	200mW (23dBm)
RFX1200	Transceiver	1150 to 1450 MHz	200mW (23dBm)
RFX1800	Transceiver	1.5 to 2.1 GHz	100mW (20dBm)
RFX2400	Transceiver	2.3 to 2.9 GHz	50mW (17dBm)
XCVR2450	Transceiver	2.4 to 2.5 GHz, and 4.9 to 5.9 GHz	100mW (20dBm)

**Table 2.** Transmitter frequency parameters (GHz).

Band	LO frequency combination	center frequency	SW position
1. 0.4 – 0.9	0.65	0.65 + *f*_0_	SW1 lower
2. 0.9 – 1.4	1.15	1.15 + *f*_0_	SW1 upper
3. 1.4 – 1.9	3.15, 1.5	1.65 + *f*_1_	SW2 lower
4. 1.9 – 2.4	3.65, 1.5	2.15 + *f*_2_	SW2 lower
5. 2.4 – 2.9	3.15, 0.5	2.65 + *f*_1_	SW2 lower
6. 2.9 – 3.4	3.65, 0.5	3.15 + *f*_2_	SW2 lower
7. 3.4 – 2.9	3.15, 0.5	3.65 + *f*_1_	SW2 upper
8. 2.9 – 4.4	3.65, 0.5	4.15 + *f*_2_	SW2 upper
9. 4.4 – 4.9	3.15, 1.5	4.65 + *f*_1_	SW2 upper
10. 4.9 – 5.4	3.65, 1.5	5.15 + *f*_2_	SW2 upper

**Table 3.** Receiver frequency parameters (GHz).

Band	Intermediate frequency (IF)	Image frequency
1. 0.4 – 0.9	0	
2. 0.9 – 1.4	0	
3. 1.4 – 2.4	3.9, 0	5.4 – 6.4
4. 2.4 – 3.4	3.9, 0	4.4 – 5.4
5. 3.4 – 4.4	3.9, 0	
6. 4.4 – 5.4	3.9, 0	2.4 – 3.4
