# Qiu, R. *et al.* A Unified Multi-Functional Dynamic Spectrum Access Framework: Tutorial, Theory and Multi-GHz Wideband Testbed. *Sensors* 2009, *9*, 6530–6603

**DOI:** 10.3390/s90906835

**Published:** 2009-08-31

**Authors:** Robert Qiu, Nan Guo, Husheng Li, Zhiqiang Wu, Vasu Chakravarthy, Yu Song, Zhen Hu, Peng Zhang, Zhe Chen

**Affiliations:** 1 Department of Electrical and Computer Engineering, Center for Manufacturing Research, Tennessee Technological University, Cookeville, TN 38505, USA; E-Mails: nguo@tntech.edu (N.G.); ysong21@tntech.edu (Y.S.); zhu21@tntech.edu (Z.H.); pzhang21@tntech.edu (P.Z.); zchen42@tntech.edu (Z.C.); 2 Department of Electrical Engineering and Computer Science, The University of Tennessee Knoxville, TN 37996-2100, USA; E-Mail: husheng@eecs.utk.edu; 3 Department of Electrical Engineering, Wright State University, 3640 Colonel Glenn Highway, Dayton, OH 45435-0001, USA; E-Mail: zhiqiang.wu@wright.edu; 4 Air Force Research Lab Sensors Directorate, Wright-Patterson Air Force Base, Dayton, OH 45433, USA; E-Mail: Vasu.Chakravarthy@wpafb.af.mil (V.C.)

We found that the affiliation of author Vasu Chakravarthy was incorrect in our paper published in *Sensors* recently [1]. Therefore Vasu Chakravarthy’s affiliation is corrected as follows: Air Force Research Lab Sensors Directorate, Wright-Patterson Air Force Base, Dayton, OH 45433, USA.
